# Prevalence, geographic distribution and risk factors of *Eimeria* species on commercial broiler farms in Guangdong, China

**DOI:** 10.1186/s12917-024-03990-4

**Published:** 2024-05-03

**Authors:** Shenquan Liao, Xuhui Lin, Qingfeng Zhou, Zhuanqiang Yan, Caiyan Wu, Juan Li, Minna Lv, Junjing Hu, Haiming Cai, Yongle Song, Xiangjie Chen, Yibin Zhu, Lijun Yin, Jianfei Zhang, Nanshan Qi, Mingfei Sun

**Affiliations:** 1 Key Laboratory of Livestock Disease Prevention of Guangdong Province, Key Laboratory of Avian Influenza and Other Major Poultry Diseases Prevention and Control, Ministry of Agriculture and Rural Affairs, Institute of Animal Health, Guangdong Academy of Agricultural Sciences, Guangzhou, China; 2 Wen’s Group Academy, Wen’s Foodstuffs Group Co., Ltd., Xinxing, Guangdong China

**Keywords:** Broiler, *Eimeria*, Prevalence, Risk factors

## Abstract

**Background:**

Coccidiosis is one of the most frequently reported diseases in chickens, causing a significant economic impact on the poultry industry. However, there have been no previous studies evaluating the prevalence of this disease in broiler farms in Guangdong province. Therefore, this study aims to conduct an epidemiological investigation into the occurrence of *Eimeria* species and associated risk factors in intensive management conditions across four regions in Guangdong province, China. A total of 394 fecal samples were collected from 89 broiler farms in Guangdong province. The prevalence of *Eimeria* species infection was determined using PCR, and the occurrence of *Clostridium perfringens* type A was assessed using quantitative real-time PCR.

**Results:**

The results showed an overall prevalence of 98.88% (88/89) at the farm level and 87.06% (343/394) at the flock level. All seven *Eimeria* species were identified, with *E. acervulina* (72.53%; 64/89), *E. tenella* (68.54%; 61/89), and *E. mitis* (66.29%; 59/89) at the farm level, and *E. acervulina* (36.55%; 144/394), *E. mitis* (35.28%; 139/394), and *E. tenella* (34.01%; 134/394) at the flock level. The predominant species combination observed was a co-infection of all seven *Eimeria* species (6.74%; 6/89), followed by a combination of *E. acervulina*, *E. tenella*, *E. mitis*, *E. necatrix*, *E. brunetti*, and *E. maxima* (5.62%, 5/89). A combination of *E. acervulina*, *E. tenella*, *E. mitis*, *E. necatrix*, *E. brunetti*, and *E. praecox* (4.49%; 4/89) was also observed at the farm level. Furthermore, the study identified several potential risk factors associated with the prevalence of *Eimeria* species, including farm location, chicken age, drinking water source, control strategy, and the presence of *C. perfringens* type A were identified as potential risk factors associated with prevalence of *Eimeria* species. Univariate and multivariate analyses revealed a significant association between *E. necatrix* infection and both grower chickens (OR = 10.86; 95% CI: 1.92–61.36; *p* < 0.05) and adult chickens (OR = 24.97; 95% CI: 4.29–145.15; *p* < 0.001) compared to starter chickens at the farm level. Additionally, farms that used groundwater (OR = 0.27; 95% CI: 0.08–0.94; *p* < 0.05) were less likely to have *E. maxima* compared to those that used running water. At the flock level, the prevalence of *E. tenella* was significantly higher in the Pearl River Delta (OR = 2.48; 95% CI: 1.0–6.15; *p* = 0.05) compared to eastern Guangdong. Interestingly, flocks with indigenous birds were less likely to have *E. brunetti* (OR = 0.48; 95% CI: 0.26–0.89; *p* < 0.05) compared to flocks with indigenous crossbred birds. Furthermore, flocks that used anticoccidial drugs (OR = 0.09; 95% CI: 0.03–0.31; *p* < 0.001) or a combination of vaccines and anticoccidial drugs (OR = 0.06; 95% CI: 0.01–0.25; *p* < 0.001) were less likely to be positive for *E. tenella* compared to flocks that only used vaccines. Finally, flocks with *C. perfringens* type A infection were significantly more likely to have *E. necatrix* (OR = 3.26; 95% CI: 1.96–5.43; *p* < 0.001), *E. tenella* (OR = 2.14; 95% CI: 1.36–3.36; *p* < 0.001), *E. brunetti* (OR = 2.48; 95% CI: 1.45–4.23; *p* < 0.001), and *E. acervulina* (OR = 2.62; 95% CI: 1.69–4.06; *p* < 0.001) compared to flocks without *C. perfringens* type A.

**Conclusions:**

This study conducted an investigation on the prevalence, distribution, and risk factors associated with *Eimeria* species infection in broiler chickens in Guangdong. The farm-level prevalence of *Eimeria* species was higher than the previous prevalence figures for other areas and countries. *E. brunetti* was identified at higher prevalence in Guangdong than previously survived prevalence in different regions in China. Farm location, chicken age, drinking water source, control strategy, and the presence of *C. perfringens* type A were considered as potential risk factors associated with prevalence of *Eimeria* species. It is imperative to underscore the necessity for further surveys to delve deeper into the occurrence of *Eimeria* species under intensive management conditions for different flock purposes.

**Supplementary Information:**

The online version contains supplementary material available at 10.1186/s12917-024-03990-4.

## Background

Coccidiosis is a highly prevalent disease that affects chickens globally. It is caused by protozoan parasites from the *Eimeria* genus and can cause significant damage to the intestinal tract. This results in increased mortality rates, reduced weight gain, impaired nutrient absorption, and heightened susceptibility to other enteric pathogens [1]. The far-reaching consequences of this disease have a profound economic impact on the poultry industry [2]. In chickens, there are seven mainly recognized species of *Eimeria*: *E. tenella*, *E. necatrix*, *E. brunetti*, *E. acervulina*, *E. maxima*, *E. mitis*, and *E. praecox*. Each of these species has a preference for specific segments of the intestinal tract and exhibits varying levels of pathogenicity, resulting in distinct clinical manifestations [3]. *E. necatrix* is considered the most pathogenic species, while *E. tenella*, is relatively prevalent and both can cause bloody lesions and high rates of morbidity and mortality in chickens [4]; *E. brunetti* is highly pathogenic and is associated with haemorrhagic coccidiosis [5]. On the other hand, *E. acervulina* and *E. maxima* are classified as moderately pathogenic, causing inflammation of the intestinal wall characterized by pinpoint haemorrhage and epithelial demolition [5]. Finally, *E. mitis* and *E. praecox* are generally considered less pathogenic, causing malabsorption and enteritis [3].

Control strategies for coccidiosis primarily rely on chemotherapy or vaccination. However, the emergence of drug resistance in various regions and the lack of new anticoccidial drugs have led to a decrease in the effectiveness of these agents [6]. In recent decades, live anticoccidial vaccines have been utilized to prevent coccidiosis [7]. Currently, there are three types of live anticoccidial vaccines currently available in China: a trivalent vaccine containing *E. tenella*, *E. acervulina* and *E. maxima*; a tetravalent vaccine containing *E. tenella*, *E. necatrix*, *E. acervulina*, and *E. maxima*; and an imported vaccine, Coccivac™, containing *E. maxima*, *E. mivati*, *E. acervulina* and *E. tenella*. In order to accurately assess the effectiveness of these control strategies, including the composition of vaccines, it is crucial to have a thorough understanding of the epidemiology of *Eimeria* species and the potential risk factors associated with the occurrence of different *Eimeria* species.

The conventional taxonomy of *Eimeria* species has traditionally relied on morphological characteristics, the affected segments of the intestinal tract, and the pre-patent period of the *Eimeria* following in vivo infection in chickens [5]. However, these methods may not always provide precise diagnoses [8]. In recent decades, polymerase chain reaction (PCR) techniques have emerged as a valuable tool for identifying all seven *Eimeria* species. This molecular method utilizes genetic markers located within the internal transcribed spacer-1 (ITS-1), ITS-2, and the sequence characterized amplified region (SCAR) [9–12]. Currently, there is a lack of accurate data and previously reported information on the prevalence of *Eimeria* species in broiler farms in Guangdong province, China. Therefore, the purpose of this study is to investigate the epidemiology of *Eimeria* species in Guangdong province and analyze the associated risk factors. The findings from this study will not only contribute to our understanding of the occurrence and potential control strategies for coccidiosis in poultry in Guangdong province, China, but also enhance our comprehension of the potential risk factors associated with intensive poultry management practices.

## Methods

### Study area and farms

**Fig. 1:**
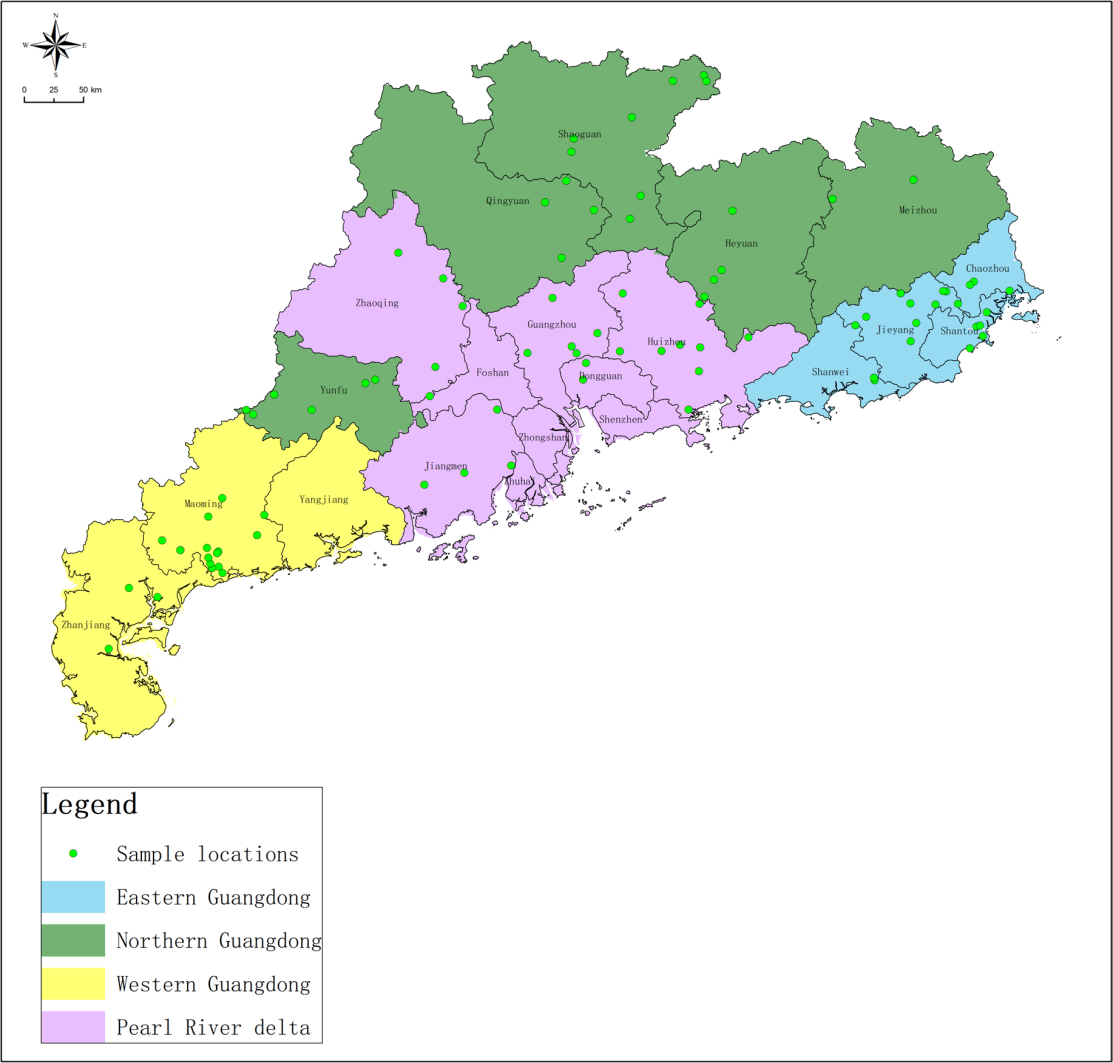
Approximate locations of 89 broiler farms included in this study. Each green dot represents an approximate farm location. Eastern, Western, Northern, and the Pearl River Delta of Guangdong are shaded as indicated

The study was conducted across four distinct regions, spanning geographically between 20°09'–25°31' north latitude and 109°45'–117°20' east longitude located in southern China. These regions covering a total land area of 179,800 km^2^. The study was carried out over an extensive timeframe, spanning from April 2020 to November 2021. The climate in Guangdong is subtropical, characterized by mild winters and hot, humid summers. The average annual temperature ranged from 23 to 25 °C. Additionally, the relative humidity levels ranged from 57 to 77% on average. The average monthly rainfall was approximately from 118 mm to 150 mm, drawing data from https://www.worldweatheronline.com/ as the source (Table 1). The selection of poultry farms depended on the number of broiler farms across four areas in Guangdong province. This study included 89 broiler farms (21 from eastern Guangdong, 19 from western Guangdong, 24 from northern Guangdong, and 25 from Pearl River Delta) (Fig. 1). Each farm had between 2 and 20 houses, with bird populations ranging from 5,000 to 40,000 individuals and a density of 10 to 16 birds/m^2^. The most common broiler breeds are the three-yellow chicken and the spotted-brown chicken. The bedding materials in use were wood shavings or rice husk.

**Table 1:** Managing characteristics of broiler farms in four regions of Guangdong, China during 2020 to 2021

Variables	Eastern Guangdong	Western Guangdong	Northern Guangdong	Peal River Delta
Annual average temperature (°C)	23.04	24.67	24.0	24.63
Annual average humidity (%)	73.08	76.75	64.17	57.88
Annual average rainfall (mm)	118.69	119.80	149.50	149.81
Genetic line in number of sampled farms^a^				
Indigenous	12	13	17	7
Indigenous crossbred	9	6	7	18
Litter composition	wood shavings	wood shavings	wood shavings/rice husk	wood shavings/rice husk
Type of farming	ground floor	ground floor	ground floor/multi-layer cage	ground floor/multi-layer cage
Type of drinking water	running water/groundwater	running water/groundwater	running water/groundwater	running water/groundwater
Average age of birds at sampling (min. to max.)	45 (20–65)	51 (23–90)	49 (22–79)	45 (17–86)
Flock size (min. to max.)	11,667 (5000–20,000)	14,975 (9000–20,000)	17,341 (7000–40,000)	11,669 (8000–23,000)

^a^Eighty-nine total surveys

### Questionnaire design

Based on previous studies collecting data on farm management, performance figures, bird characteristics, chicken health and social factors, a questionnaire was developed for analyzing risk factors in this study to identify risk factors associated with *Eimeria* species distribution (Supplemental Table S1) [13]. The questionnaire for broiler farmers and/or veterinarians included 21 questions. In detail, the survey gathered information on bird-related factors (e.g., age, breed, flock size, and flock density), along with flock management practices associated with coccidiosis, such as general information on the farm (e.g., farm location, type of production, type of farming, litter composition, source of drinking water, and fecal treatment method), data regarding coccidiosis occurrence (e.g., coccidiosis detection, *Eimeria* species identification), and strategies for coccidiosis control (e.g., the use of coccidiostats and/or vaccines) (Supplemental Tables S2 and S3).

### Fecal sample collection and sample analysis

Broiler flocks were sampled for this study according to the scale of poultry operations on the farm. On small-scale broiler farms, between 1 and 4 flocks were sampled, whereas on large-scale broiler farms, sampling involved 5 to 16 flocks. A total of 394 fecal samples were obtained on 89 farms. For sample collection, fresh fecal samples were obtained from different sites in each poultry house, as previously described by Kumar et al. [14]. This method included tracing a W-shaped pattern along each poultry house. Each sample, weighed approximately 250 g, was made up of 30 fresh fecal droppings collected from a single house. Samples were placed in labelled zipped plastic bags and immediately transported at 4 °C to the laboratory. Each sample was mixed with an equal volume of sterile ddH_2_O and was homogenized using a blender. 200 µl aliquots of the prepared samples were transferred into a 1.5 ml Eppendorf tubes for DNA extraction. The E.Z.N.A.® Stool DNA Kit (Omega, D4015) was used for genomic DNA extraction, following the manufacturer’s protocol. The extracted DNA was then stored at -20 °C until further use.

PCR was performed separately for each *Eimeria* species. The primer sequences for each *Eimeria* species can be found in Table 2, as previously described by Schnitzler et al. [15, 16] and Haug et al. [10]. Each amplification reaction consisted of a total volume of 20 µl, including 10 µl of Premix *Taq*™ (Takara, RR901A), 500 nm of species-specific for forward and reverse primers, 2 µl of DNA sample, and 6 µl of ddH_2_O. The amplification was carried out using a T100™ thermal cycler (Bio-Rad, USA) with the following cycling conditions: an initial denaturation step at 95 °C for 2 min, followed by 35 repeat cycles, each consisting of 30 s of denaturation at 95 °C, 30 s at 62 °C for annealing, and 1 min at 72 ° for extension, with a final extension step of 3 min. The resulting amplification products were then analyzed by electrophoresis using a 1.5% agarose gel (Supplemental Figure S1).

**Table 2:** Primers used for the detection of seven chicken *Eimeria* species

* Eimeria* species	Primer	Sequence 5′ to 3′	Annealing temperature (°C)	Amplicon size (bp)	References
*Eimeria necatrix*	ENF	TACATCCCAATCTTTGAATCG	61	383	Schnitzler et al. 1998 [15]
	ENR	GGCATACTAGCTTCGAGCAAC			
*Eimeria tenella*	ETF	AATTTAGTCCATCGCAACCCT	60	271	Schnitzler et al. 1998 [15]
	ETR	CGAGCGCTCTGCATACGACA			
*Eimeria brunetti*	EBF	GATCAGTTTGAGCAAACCTTCG	45	310	Schnitzler et al. 1998 [15]
	EBR	TGGTCTTCCGTACGTCGGAT			
*Eimeria acervulina*	EAF	GGCTTGGATGATGTTTGCTG	60	321	Schnitzler et al. 1998 [15]
	EAR	CGAACGCAATAACACACGCT			
*Eimeria maxima*	EmuF	GTGGGACTGTGGTGATGGGG	60	162	Haug et al. 2007 [10]
	EmuR	ACCAGCATGCGCTCACAACCC			
*Eimeria mitis*	EMIF	TATTTCCTGTCGTCGTCTCGC	54	306	Schnitzler et al. 1999 [16]
	EMIR	GTATGCAAGAGAGAATCGGGA			
*Eimeria praecox*	EPF	CATCATCGGAATGGCTTTTTGA	54	368	Schnitzler et al. 1999 [16]
	EPR	AATAAATAGCGCAAAATTAAGCA			

The identification of *C. perfringens* type A in fecal samples was conducted using quantitative real-time PCR (qPCR) targeting the alpha toxin gene, as described by Mohiuddin et al. [17]. The qPCR was carried out in a reaction mixture of 20 ul, containing TB Green *Premix Ex Taq* II (Takara, RR820B) (10 µL), forward primers (1 µL), reverse primers (1 µL), template DNA 1µL (150–200 ng), and ddH_2_O (7 µL). The amplification process was performed using CFX Connect™ Real-Time PCR System (Bio-Rad, USA). The amplification program was at 95 °C for 30 s, 35 cycles of denaturation at 95 °C for 15 s, annealing at 60 °C for 30 s, and a final step for dissociation at 95 °C for 10 s, 65 °C for 5 s, and 95 °C for 5 s.

### Statistical analysis

All statistical analyses were performed using software IBM SPSS Statistics 27.0 software (SPSS Inc., http://www.spss.com.hk). Descriptive statistics, including bird age, bird breed, flock size, farming type, type of drinking water, and control strategy were obtained from the questionnaires. The prevalence of *Eimeria* spp. infections, with a 95% confidence interval (CI), was initially calculated. Univariable and multivariable logistic regression models were then used to assess the predictor variables associated with the presence of *Eimeria* species. Multivariable models were built using forward stepwise logistic regression procedures, with inclusion if *p* < 0.05. The prevalence of each species of *Eimeria* infection in variables such as age, breed, flock size, farming type, drinking water source, control strategy, region, and presence of *C. perfringens* type A was compared using chi-square test or Fisher’s exact test. The odds ratio (OR) with a 95% CI was calculated to assess the associations between participants’ characteristics and *Eimeria* species infection. A *p* value of ≤ 0.05 was considered as statistically significant.

## Results

### Infection of *Eimeria* species in broiler chickens in Guangdong

An epidemiological study was conducted in Guangdong province from April 2020 to November 2021 to investigate the prevalence of *Eimeria* species infection in broiler chickens. A total of 394 flocks from 89 broiler farms were examined for the presence of *Eimeria* species. The overall farm-level infection rate was 98.88% (88/89; 95% CI: 96.64–100%), while the flock-level prevalence was 87.06% (343/394; 95% CI: 83.73–90.38%) (Table 3). All four regions of Guangdong were found to have seven *Eimeria* species present. The most common species at the farm-level were *E. acervulina* (72.53%; 64/89; 95% CI: 63.18–81.88%), *E. tenella* (68.54%; 61/89; 95% CI: 58.70–78.38%), *E. mitis* (66.29%; 59/89; 95% CI: 56.28–76.31%), and *E. necatrix* (61.80%; 55/89; 95% CI: 51.50–72.09%). At the flock-level, the predominant species were *E. acervulina* (36.55%; 144/394; 95% CI: 31.77–41.32%), *E. mitis* (35.28%; 139/394; 95% CI: 30.54–40.02%), *E. tenella* (34.01%; 134/394; 95% CI: 29.31–38.71%), and *E. necatrix* (30.96%; 122/394; 95% CI: 26.38–35.55%). Geographically, *E. necatrix* was significantly more prevalent in northern Guangdong (87.50%; 21/24; 95% CI: 73.23–100%) at the farm-level (*p* < 0.05), as well as at the flock-level with a prevalence of 46.77% in northern Guangdong (58/124; 95% CI: 37.87–55.68%) (*p* < 0.001). In contrast, *E. acervulina* was more prevalent in both eastern (47.13%; 41/87; 95% CI: 36.43–57.83%) and western Guangdong (45.71%; 32/70; 95% CI: 33.75–57.68%) at the flock-level (*p* < 0.05). Additionally, both *E. tenella* and *E. acervulina* were more prevalent in eastern Guangdong, with a prevalence of 45.98% (40/87; 95% CI: 35.29–56.66%) (*p* < 0.05), and 47.13% (41/87; 95% CI: 36.43–57.83%) (*p* < 0.05), respectively (Table 3).

**Table 3:** Farm-level and flock-level prevalence of seven avian *Eimeria* species in broiler chickens from Guangdong province, China

* Eimeria* species	Category	Eastern Guangdong (*n*^a^=21, *n*^b^=87)		Western Guangdong (*n*^a^=19, *n*^b^=70)		Northern Guangdong (*n*^a^=24, *n*^b^=124)		Peal River Delta (*n*^a^=25, *n*^b^=113)		All regions (*n*^a^=89, *n*^b^=394)		* P*-value
		No. positive	Prevalence (95% CI)	No. positive	Prevalence (95% CI)	No. positive	Prevalence (95% CI)	No. positive	Prevalence (95% CI)	No. positive	Prevalence (95% CI)	
*Eimeria necatrix*	Farm-level	8	38.10 (15.44–60.75)	10	52.63 (27.91–77.36)	21	**87.50 (73.23–100.0)**	16	64.0 (43.78–84.22)	55	61.80 (51.50-72.09)	**0.006**
	Flock-level	12	13.79 (6.40-21.19)	16	22.86 (12.77–32.94)	58	**46.77 (37.87–55.68)**	36	31.86 (23.14–40.58)	122	30.96 (26.38–35.55)	**< 0.001**
*Eimeria tenella*	Farm-level	16	76.19 (56.32–96.06)	12	63.16 (39.27–87.04)	17	70.83 (51.23–90.44)	16	64.0 (43.78–84.22)	61	68.54 (58.70-78.38)	0.772
	Flock-level	40	**45.98 (35.29–56.66)**	17	24.29 (13.99–34.58)	38	30.65 (22.42–38.87)	39	34.51 (25.61–43.41)	134	34.01 (29.31–38.71)	**0.027**
*Eimeria brunetti*	Farm-level	9	42.86 (19.77–65.94)	10	52.63 (27.91–77.36)	14	58.33 (37.07–79.60)	14	56.0 (35.09–76.91)	47	52.81 (42.23–63.38)	0.746
	Flock-level	19	21.84 (12.98–30.70)	12	17.14 (8.09–26.19)	22	17.74 (10.92–24.56)	25	22.12 (14.35–29.90)	78	19.80 (15.85–23.75)	0.740
*Eimeria acervulina*	Farm-level	14	66.67 (44.68–88.65)	17	89.47 (74.28–100.0)	15	62.50 (41.61–83.38)	18	72.0 (53.08–90.92)	64	72.53 (63.18–81.88)	0.237
	Flock-level	41	**47.13 (36.43–57.83)**	32	**45.71 (33.75–57.68)**	31	25.0 (17.27–32.73)	40	35.40 (26.45–44.35)	144	36.55 (31.77–41.32)	**0.003**
*Eimeria maxima*	Farm-level	10	47.62 (24.32–70.91)	11	57.89 (33.45–82.34)	11	45.83 (24.34–67.33)	8	32.0 (12.35–51.65)	40	44.94 (34.41–55.48)	0.384
	Flock-level	24	27.59 (18.01–37.17)	12	17.14 (8.09–26.19)	23	18.55 (11.61–25.49)	15	13.27 (6.92–19.63)	74	18.78 (14.91–22.66)	0.079
*Eimeria mitis*	Farm-level	12	57.14 (34.06–80.23)	13	68.42 (45.40-91.44)	16	66.67 (46.33-87.0)	18	72.0 (53.08–90.92)	59	66.29 (56.28–76.31)	0.755
	Flock-level	30	34.48 (24.29–44.67)	22	31.43 (20.28–42.58)	47	37.90 (29.24–46.56)	40	35.40 (26.45–44.35)	139	35.28 (30.54–40.02)	0.837
*Eimeria praecox*	Farm-level	8	38.10 (15.44–60.75)	7	36.84 (12.96–60.73)	10	41.67 (20.40-62.93)	12	48.0 (26.95–69.05)	37	41.57 (31.13–52.01)	0.872
	Flock-level	21	24.14 (14.96–33.31)	7	10.0 (2.80–17.20)	17	13.71 (7.59–19.85)	21	18.58 (11.30-25.87)	66	16.75 (13.05–20.45)	0.079
Overall	Farm-level	21	100.0 (—)	19	100.0 (—)	24	100.0 (—)	24	96.0 (87.74–100.0)	88	98.88 (96.64–100.0)	0.459
	Flock-level	81	93.10 (87.67–98.54)	63	90.0 (82.80–97.20)	107	86.29 (80.15–92.43)	92	81.42 (74.13–88.70)	343	87.06 (83.73–90.38)	0.085

n^a^ – total number of farms, n^b^ – total number of flocks, significant predictors in bold

*95% CI* 95% confidence interval

### Mixed infection of *Eimeria* species

In this study, the prevalence of infection with two or more *Eimeria* species was found to be 93.25% (83/89) at the farm level and 49.75% (196/394) at the flock level. Co-infection with three and four *Eimeria* species was more common among the 89 farms included, with a proportion of 20.22% (18/89; 95% CI: 11.72–28.73%) for both, followed by co-infection with five *Eimeria* species, which was found in 17.95% (16/89; 95% CI: 9.84–26.11%) of the farms. In terms of single-species infections at the flock level, they were prevalent across all four regions of Guangdong, with a proportion of 37.31% (147/394; 95% CI: 32.51–42.11%). This was followed by co-infection with two *Eimeria* species, which was found in 19.80% (78/394; 95% CI: 15.85–23.75%) of the flocks. Only 49.75% (196/394) of samples contained two or more *Eimeria* species within a single fecal sample at the flock level (Table 4). At the farm level, the most common combination of *Eimeria* species was all seven species (6.74%; 6/89), followed by *E. acervulina*, *E. tenella*, *E. mitis*, *E. necatrix*, *E. brunetti*, and *E. maxima* (5.62%, 5/89), and *E. acervulina*, *E. tenella*, *E. mitis*, *E. necatrix*, *E. brunetti*, and *E. praecox* (4.49%; 4/89) (Table 5).

**Table 4:** Farm-level and flock-level frequency of mixed infections in broiler chickens from Guangdong province, China

No. species	Farm-level (*n*^a^=89)		Flock-level (*n*^b^=394)	
	No. positive	Proportion (95% CI)	No. positive	Prevalence (95% CI)
One	5	5.62 (0.74–10.50)	147	37.31 (32.51–42.11)
Two	10	11.24 (4.55–17.93)	78	19.80 (15.85–23.75)
Three	18	20.22 (11.72–28.73)	59	14.97 (11.44–18.51)
Four	18	20.22 (11.72–28.73)	29	7.36 (4.77–9.95)
Five	16	17.98 (9.84–26.11)	21	5.33 (3.10–7.56)
Six	15	16.85 (8.92–24.78)	7	1.78 (0.47–3.09)
Seven	6	6.74 (1.43–12.05)	2	0.51 (0-1.21)

n^a^ – total number of farms, n^b^ – total number of flocks

*95% CI* 95% confidence interval

**Table 5:** Diversity and distribution of *Eimeria* species in broiler farms from Guangdong province, China

* Eimeria* species							Number of species	Number of positive farms (*n* = 89)	Proportion (%)
*E. acervulina*	*E. tenella*	*E. mitis*	*E. necatrix*	*E. brunetti*	*E. maxima*	*E. praecox*			
+	+	+	+	+	+	+	7	6	6.74
+	+	+	+	+	+	–	6	5	5.62
+	+	+	+	+	–	+	6	4	4.49
–	+	+	+	+	–	+	5	3	3.37
+	+	+	–	–	–	+	4	3	3.37
+	+	–	+	–	+	–	4	3	3.37
+	–	–	+	+	–	–	3	3	3.37
+	+	+	+	–	+	+	6	2	2.25
+	+	–	+	+	+	+	6	2	2.25
+	–	+	+	+	–	+	5	2	2.25
+	–	+	+	+	+	–	5	2	2.25
+	+	+	+	–	+	–	5	2	2.25
–	+	+	+	+	+	–	5	2	2.25
+	+	+	+	+	–	+	4	2	2.25
+	+	+	+	–	–	–	4	2	2.25
–	+	+	+	–	–	+	4	2	2.25
+	+	+	–	–	–	–	3	2	2.25
+	–	+	–	–	+	–	3	2	2.25
+	+	–	–	–	–	–	2	2	2.25
–	–	+	+	–	–	–	2	2	2.25
–	+	–	+	–	–	–	2	2	2.25
–	+	–	–	–	–	–	1	2	2.25
+	+	+	–	+	+	+	6	1	1.12
–	+	+	+	+	+	+	6	1	1.12
–	–	+	+	+	+	+	5	1	1.12
+	+	–	+	+	+	–	5	1	1.12
+	–	+	+	–	+	+	5	1	1.12
+	+	+	–	+	–	+	5	1	1.12
+	+	+	–	–	+	+	5	1	1.12
+	+	+	–	–	+	–	4	1	1.12
+	+	–	–	+	+	–	4	1	1.12
+	+	–	+	–	–	+	4	1	1.12
+	–	+	+	–	–	+	4	1	1.12
+	–	+	–	+	+	–	4	1	1.12
–	+	–	+	+	+	–	4	1	1.12
+	–	+	–	–	–	+	3	1	1.12
+	+	–	–	–	+	–	3	1	1.12
+	+	–	–	–	–	+	3	1	1.12
+	–	+	–	+	–	–	3	1	1.12
+	+	–	–	+	–	–	3	1	1.12
+	–	–	–	+	–	+	3	1	1.12
+	–	+	+	–	–	–	3	1	1.12
+	+	–	+	–	–	–	3	1	1.12
–	–	+	+	+	–	–	3	1	1.12
–	+	+	+	–	–	–	3	1	1.12
–	+	–	–	+	+	–	3	1	1.12
+	–	–	–	+	–	–	2	1	1.12
+	–	+	–	–	–	–	2	1	1.12
–	+	–	–	–	+	–	2	1	1.12
–	+	–	–	+	–	–	2	1	1.12
–	–	+	–	–	–	–	1	1	1.12
–	–	–	+	–	–	–	1	1	1.12
–	–	–	–	–	+	–	1	1	1.12

### Risk factors associated with *Eimeria* species infection

Univariate analysis was performed to determine the associations between the prevalence of *Eimeria* species infection at the farm level and various factors, such as farm location, bird age, drinking water source, control strategy, and presence of *C. perfringens* type A (Table 6). Multivariate analysis at the farm level revealed significant associations between *E. necatrix* infection and both grower birds (OR = 10.86; 95% CI: 1.92–61.36; *p* < 0.05) and adult birds (OR = 24.97; 95% CI: 4.29–145.15; *p* < 0.001) compared to starter birds. Additionally, a significant positive association was found between *E. brunetti* infection and adult chickens (OR = 5.02; 95% CI: 1.41–17.83; *p* < 0.05) compared to starter chickens. Farms that used groundwater (OR = 0.27; 95% CI: 0.08–0.94; *p* < 0.05) were less likely to have *E. maxima* compared to farms that used running water. Furthermore, farms with *C. perfringens* type A infection showed a significant positive association with *E. brunetti* (OR = 6.53; 95% CI: 1.52–28.09; *p* < 0.05), *E. acervulina* (OR = 5.30; 95% CI: 1.41–19.95; *p* < 0.05), *E. mitis* (OR = 4.23; 95% CI: 1.17–15.33; *p* < 0.05), and *E. praecox* (OR = 7.63; 95% CI: 1.45–40.09; *p* < 0.05) infections compared to farms without *C. perfringens* type A detected (Table 7).

**Table 6:** Univariate analysis of putative farm-level risk factors associated with *Eimeria* species infection in broiler chickens from Guangdong province, China

Variables	*E. necatrix*		*E. tenella*		*E. brunetti*		*E. acervulina*		*E. maxima*		*E. mitis*		*E. praecox*	
	OR (95% CI)	* P*-value	OR (95% CI)	* P*-value	OR (95% CI)	* P*-value	OR (95% CI)	* P*-value	OR (95% CI)	* P*-value	OR (95% CI)	* P*-value	OR (95% CI)	* P*-value
Region														
Eastern (*n* = 21)	Reference	—	Reference	—	Reference	—	Reference	—	Reference	—	Reference	—	Reference	—
Western (*n* = 19)	1.81 (0.51–6.36)	0.358	0.54 (0.14–2.11)	0.372	1.48 (0.43–5.16)	0.537	4.25 (0.76–23.81)	0.100	1.51 (0.43–5.28)	0.517	1.63 (0.44–5.95)	0.463	0.95 (0.26–3.42)	0.935
Northern (*n* = 24)	**11.38 (2.55–50.79)**	**0.001**	0.76 (0.20–2.89)	0.686	1.87 (0.57–6.11)	0.302	0.83 (0.24–2.84)	0.771	0.93 (0.29–3.01)	0.905	1.50 (0.45–5.04)	0.512	1.16 (0.35–3.84)	0.807
Pearl River Delta (*n* = 25)	2.89 (0.87–9.60)	0.083	0.56 (0.15–2.03)	0.373	1.70 (0.53–5.47)	0.376	1.29 (0.37–4.53)	0.696	0.52 (0.16–1.72)	0.282	1.93 (0.57–6.59)	0.295	1.50 (0.46–4.88)	0.500
Age (weeks)														
Starter (0–4, *n* = 25)	Reference	—	Reference	—	Reference	—	Reference	—	Reference	—	Reference	—	Reference	—
Grower (4–8, *n* = 29)	**6.75 (2.05–22.27)**	**0.002**	1.22 (0.36–4.14)	0.747	2.19 (0.73–6.55)	0.162	1.22 (0.36–4.14)	0.747	1.66 (0.56–4.96)	0.364	1.75 (0.57–5.32)	0.327	2.40 (0.77–7.48)	0.131
Adult (> 8, *n* = 35)	**8.68 (2.68–28.15)**	**< 0.001**	0.58 (0.19–1.76)	0.339	**3.01 (1.04–8.74)**	**0.043**	0.85 (0.28–2.62)	0.775	1.68 (0.59–4.81)	0.334	1.96 (0.67–5.77)	0.220	2.17 (0.72–6.49)	0.168
Breed														
Indigenous crossbred (*n* = 40)	Reference	—	Reference	—	Reference	—	Reference	—	Reference	—	Reference	—	Reference	—
Indigenous (*n* = 49)	0.95 (0.40–2.24)	0.902	1.09 (0.44–2.68)	0.849	0.49 (0.21–1.15)	0.100	1.19 (0.47–3.07)	0.717	1.20 (0.52–2.78)	0.676	0.74 (0.30–1.80)	0.504	0.77 (0.33–1.81)	0.554
Flock size														
≤ 10,000 (*n* = 34)	Reference	—	Reference	—	Reference	—	Reference	—	Reference	—	Reference	—	Reference	—
> 10,000 (*n* = 55)	2.24 (0.93–5.40)	0.074	1.33 (0.53–3.31)	0.541	0.99 (0.42–2.34)	0.984	0.53 (0.20–1.46)	0.219	0.59 (0.25–1.40)	0.235	1.12 (0.46–2.76)	0.803	0.70 (0.29–1.65)	0.410
Farming														
Multi-layer cage (*n* = 9)	Reference	—	Reference	—	Reference	—	Reference	—	Reference	—	—	—	Reference	—
Ground floor (*n* = 80)	0.43 (0.08–2.20)	0.309	1.10 (0.25–4.76)	0.898	1.45 (0.36–5.81)	0.598	2.25 (0.55–9.17)	0.259	7.61 (0.91–63.69)	0.061	—	—	0.53 (0.13–2.14)	0.375
Drinking water														
Running water (*n* = 39)	Reference	—	Reference	—	Reference	—	Reference	—	Reference	—	Reference	—	Reference	—
Groundwater (*n* = 50)	**2.71 (1.12–6.53)**	**0.027**	0.94 (0.38–2.33)	0.901	1.12 (0.48–2.58)	0.799	1.58 (0.63–4.01)	0.333	0.44 (0.19–1.02)	0.057	**2.71 (1.10–6.70)**	**0.030**	2.25 (0.94–5.41)	0.070
Control														
Vaccines (*n* = 10)	Reference	—	Reference	—	Reference	—	Reference	—	Reference	—	Reference	—	Reference	—
Drugs (*n* = 46)	0.92 (0.23–3.60)	0.901	0.21 (0.02–1.79)	0.153	2.14 (0.49–9.31)	0.311	2.12 (0.51–8.91)	0.304	0.39 (0.10–1.58)	0.188	3.10 (0.76–12.66)	0.115	1.96 (0.45–8.54)	0.370
Combined strategies (*n* = 33)	**5.60 (1.17–26.72)**	**0.031**	0.22 (0.03–1.98)	0.178	**4.67 (1.01–21.64)**	**0.049**	1.53 (0.35–6.65)	0.568	0.71 (0.17–2.98)	0.638	4.0 (0.91–17.56)	0.066	1.52 (0.33–6.95)	0.592
Presence of *C. perfringens*														
No (*n* = 17)	Reference	—	Reference	—	Reference	—	Reference	—	Reference	—	Reference	—	Reference	—
Yes (*n* = 72)	2.86 (0.97–8.44)	0.057	1.24 (0.41–3.78)	0.705	**4.82 (1.43–16.25)**	**0.011**	**5.43 (1.77–16.65)**	**0.003**	2.27 (0.73–7.11)	0.159	**5.11 (1.66–15.74)**	**0.004**	**7.10 (1.51–33.30)**	**0.013**

n – total number of samples, significant predictors in bold

*95% CI* 95% confidence interval, *OR* Odds ratio

**Table 7:** Multivariate analysis of putative farm-level risk factors associated with *Eimeria* species infection in broiler chickens from Guangdong province, China

Variables	*E. necatrix*		*E. tenella*		*E. brunetti*		*E. acervulina*		*E. maxima*		*E. mitis*		*E. praecox*	
	OR (95% CI)	* P*-value	OR (95% CI)	* P*-value	OR (95% CI)	* P*-value	OR (95% CI)	* P*-value	OR (95% CI)	* P*-value	OR (95% CI)	* P*-value	OR (95% CI)	* P*-value
Region														
Eastern (*n* = 21)	Reference	—	Reference	—	Reference	—	Reference	—	Reference	—	Reference	—	Reference	—
Western (*n* = 19)	3.06 (0.39–23.99)	0.288	1.29 (0.23–7.13)	0.770	0.40 (0.07–2.22)	0.294	6.84 (0.66–70.86)	0.107	1.23 (0.23–6.61)	0.813	0.86 (0.14–5.45)	0.874	0.99 (0.18–5.50)	0.990
Northern (*n* = 24)	**59.79 (3.60-993.49)**	**0.004**	2.31 (0.36–14.98)	0.379	0.90 (0.14–5.83)	0.911	1.49 (0.19–11.67)	0.705	1.24 (0.20–7.71)	0.817	0.77 (0.11–5.66)	0.797	1.98 (0.31–12.53)	0.466
Pearl River Delta (*n* = 25)	5.22 (0.77–35.45)	0.091	1.26 (0.26–6.02)	0.775	0.77 (0.16–3.80)	0.749	1.41 (0.24–8.27)	0.707	0.63 (0.13–3.14)	0.576	0.85 (0.15–4.94)	0.860	1.50 (0.31–7.26)	0.614
Age (weeks)														
Starter (0–4, *n* = 25)	Reference	—	Reference	—	Reference	—	Reference	—	Reference	—	Reference	—	Reference	—
Grower (4–8, *n* = 29)	**10.86 (1.92–61.36)**	**0.007**	1.14 (0.29–4.55)	0.849	2.74 (0.72–10.37)	0.138	0.60 (0.13–2.79)	0.514	2.32 (0.60–8.95)	0.223	1.01 (0.26–3.98)	0.988	1.83 (0.49–6.77)	0.366
Adult (> 8, *n* = 35)	**24.97 (4.29-145.15)**	**< 0.001**	0.50 (0.16–1.61)	0.243	**5.02 (1.41–17.83)**	**0.013**	0.55 (0.14–2.10)	0.380	2.0 (0.61–6.52)	0.251	1.97 (0.57–6.72)	0.282	2.13 (0.64–7.07)	0.218
Drinking water														
Running water (*n* = 39)	Reference	—	Reference	—	Reference	—	Reference	—	Reference	—	Reference	—	Reference	—
Groundwater (*n* = 50)	1.57 (0.35–7.05)	0.556	1.07 (0.33–3.49)	0.906	0.29 (0.08–1.12)	0.072	2.04 (0.52–8.06)	0.310	**0.27 (0.08–0.94)**	**0.040**	1.81 (0.51–6.40)	0.356	1.37 (0.40–4.67)	0.618
Control														
Vaccines (*n* = 10)	Reference	—	Reference	—	Reference	—	Reference	—	Reference	—	Reference	—	Reference	—
Drugs (*n* = 46)	0.20 (0.02–2.57)	0.217	0.13 (0.01–1.73)	0.122	7.63 (0.79–73.40)	0.078	0.58 (0.06–5.19)	0.623	0.72 (0.09–5.78)	0.753	2.33 (0.26–20.95)	0.450	1.16 (0.13–10.55)	0.892
Combined strategies (*n* = 33)	0.66 (0.03–12.45)	0.778	0.09 (0.01–1.62)	0.102	**16.71 (1.25-223.23)**	**0.033**	0.30 (0.02–4.76)	0.396	1.23 (0.10–14.50)	0.872	2.86 (0.23–35.85)	0.417	0.57 (0.05–6.87)	0.657
Presence of *C. perfringens*														
No (*n* = 17)	Reference	—	Reference	—	Reference	—	Reference	—	Reference	—	Reference	—	Reference	—
Yes (*n* = 72)	5.41 (0.97–30.19)	0.054	1.64 (0.44–6.07)	0.463	**6.53 (1.52–28.09)**	**0.012**	**5.30 (1.41–19.95)**	**0.014**	3.24 (0.88-12.0)	0.078	**4.23 (1.17–15.33)**	**0.028**	**7.63 (1.45–40.09)**	**0.016**

n – total number of samples, significant predictors in bold

*95% CI* 95% confidence interval, *OR* Odds ratio

In the flock-level analysis, univariate analysis revealed significant associations between *Eimeria* species infections and several variables, including farm location, bird age, bird breed, farming practices, drinking water source, control strategy, and occurrence of *C. perfringens* type A (Table 8). Multivariate analysis showed that the prevalence of *E. tenella* was significantly higher in the Pearl River Delta (OR = 2.48; 95% CI: 1.0–6.15; *p* = 0.05) compared to eastern Guangdong. Flocks between 4 and 8 weeks of age were significantly associated with *E. brunetti* (OR = 2.63; 95% CI: 1.15–6.04; *p* < 0.05), *E. maxima* (OR = 3.05; 95% CI: 1.23–7.59; *p* < 0.05), *E. mitis* (OR = 2.01; 95% CI: 1.08–3.73; *p* < 0.05), and *E. praecox* (OR = 3.52; 95% CI: 1.44–8.62; *p* < 0.05) infections compared to flocks younger than 4 weeks flocks. Additionally, flocks older than 8 weeks were more likely to be positive for *E. necatrix* (OR = 9.65; 95% CI: 4.45–20.94; *p* < 0.001), *E. brunetti* (OR = 2.91; 95% CI: 1.31–6.44; *p* < 0.05), and *E. maxima* (OR = 2.88; 95% CI: 1.23–6.77; *p* < 0.05) infections compared to flocks younger than 4 weeks. Interestingly, flocks with indigenous birds were less likely to be positive for *E. brunetti* (OR = 0.48; 95% CI: 0.26–0.89; *p* < 0.05) compared to indigenous crossbred birds. Additionally, ground-floored flocks had a significantly higher prevalence of *E. acervulina* (OR = 2.63; 95% CI: 1.03–6.74; *p* < 0.05) compared to multi-layer caged flocks. On the other hand, ground-floored flocks were less likely to be positive for *E. necatrix* (OR = 0.34; 95% CI: 0.13–0.90; *p* < 0.05) compared to multi-layer caged flocks. Flocks treated with anticoccidial drugs (OR = 0.09; 95% CI: 0.03–0.31; *p* < 0.001) or a combination of vaccines and anticoccidial drugs (OR = 0.06; 95% CI: 0.01–0.25; *p* < 0.001) were less likely to be positive for *E. tenella* infection compared to flocks immunized with vaccines only. Flocks with *C. perfringens* type A infection had a significantly higher likelihood of being positive for *E. necatrix* (OR = 3.26; 95% CI: 1.96–5.43; *p* < 0.001), *E. tenella* (OR = 2.14; 95% CI: 1.36–3.36; *p* < 0.001), *E. brunetti* (OR = 2.48; 95% CI: 1.45–4.23; *p* < 0.001), and *E. acervulina* (OR = 2.62; 95% CI: 1.69–4.06; *p* < 0.001) infections compared to flocks that *C. perfringens* type A was not detected (Table 9).

**Table 8:** Univariate analysis of putative flock-level risk factors associated with *Eimeria* species infection in broiler chickens from Guangdong province, China

Variables	*E. necatrix*		*E. tenella*		*E. brunetti*		*E. acervulina*		*E. maxima*		*E. mitis*		*E. praecox*	
	OR (95% CI)	* P*-value	OR (95% CI)	* P*-value	OR (95% CI)	* P*-value	OR (95% CI)	* P*-value	OR (95% CI)	* P*-value	OR (95% CI)	* P*-value	OR (95% CI)	* P*-value
Region														
Eastern (*n* = 87)	Reference	—	Reference	—	Reference	—	Reference	—	Reference	—	Reference	—	Reference	—
Western (*n* = 70)	1.85 (0.81–4.23)	0.144	**0.38 (0.19–0.75)**	**0.006**	0.74 (0.33–1.65)	0.463	0.95 (0.50–1.78)	0.860	0.54 (0.25–1.18)	0.125	0.87 (0.45–1.70)	0.686	**0.35 (0.14–0.88)**	**0.025**
Northern (*n* = 124)	**5.49 (2.72–11.11)**	**< 0.001**	**0.52 (0.29–0.92)**	**0.024**	0.77 (0.39–1.53)	0.460	**0.37 (0.21–0.67)**	**< 0.001**	0.60 (0.31–1.15)	0.122	1.16 (0.66–2.06)	0.612	0.20 (0.25–1.02)	0.055
Pearl River Delta (*n* = 113)	**2.92 (1.41–6.04)**	**0.004**	0.62 (0.35–1.10)	0.101	1.02 (0.52-2.0)	0.962	0.62 (0.35–1.09)	0.095	**0.40 (0.20–0.82)**	**0.013**	1.04 (0.58–1.87)	0.893	0.72 (0.36–1.42)	0.340
Age (weeks)														
Starter (0–4, *n* = 96)	Reference	—	Reference	—	Reference	—	Reference	—	Reference	—	Reference	—	Reference	—
Grower (4–8, *n* = 150)	**2.21 (1.09–4.50)**	**0.029**	1.27 (0.74–2.20)	0.384	**2.33 (1.09-5.0)**	**0.029**	0.73 (0.43–1.24)	0.246	**2.62 (1.19–5.77)**	**0.017**	**2.11 (1.20–3.72)**	**0.009**	**3.22 (1.42–7.31)**	**0.005**
Adult (> 8, *n* = 148)	**7.0 (3.53–13.89)**	**< 0.001**	1.09 (0.63–1.86)	0.762	**2.76 (1.30–5.88)**	**0.008**	0.86 (0.51–1.46)	0.587	**2.77 (1.26–6.10)**	**0.011**	1.67 (0.95–2.96)	0.077	2.13 (0.91–4.96)	0.080
Breed														
Indigenous crossbred (*n* = 190)	Reference	—	Reference	—	Reference	—	Reference	—	Reference	—	Reference	—	Reference	—
Indigenous (*n* = 204)	1.14 (0.75–1.76)	0.537	0.98 (0.65–1.49)	0.935	**0.55 (0.33–0.90)**	**0.018**	0.78 (0.52–1.18)	0.245	0.92 (0.55–1.52)	0.734	0.88 (0.58–1.33)	0.531	0.74 (0.43–1.25)	0.261
Flock size														
≤ 10,000 (*n* = 165)	Reference	—	Reference	—	Reference	—	Reference	—	Reference	—	Reference	—	Reference	—
> 10,000 (*n* = 229)	1.42 (0.92–2.21)	0.118	0.92 (0.60–1.40)	0.685	1.19 (0.72–1.98)	0.495	0.97 (0.64–1.47)	0.883	0.87 (0.52–1.45)	0.599	1.27 (0.83–1.94)	0.266	0.67 (0.40–1.15)	0.144
Farming														
Multi-layer cage (*n* = 50)	Reference	—	Reference	—	Reference	—	Reference	—	Reference	—	Reference	—	Reference	—
Ground floor (*n* = 344)	**0.39 (0.22–0.72)**	**0.002**	1.23 (0.65–2.35)	0.552	1.34 (0.60–2.99)	0.472	1.97 (0.99–3.91)	0.052	**6.35 (1.51–26.76)**	**0.012**	0.79 (0.43–1.46)	0.455	1.07 (0.48–2.39)	0.879
Drinking water														
Running water (*n* = 139)	Reference	—	Reference	—	Reference	—	Reference	—	Reference	—	Reference	—	Reference	—
Groundwater (*n* = 255)	**1.82 (1.14–2.92)**	**0.012**	**0.59 (0.39–0.91)**	**0.017**	**0.56 (0.34–0.93)**	**0.026**	0.99 (0.65–1.52)	0.965	**0.58 (0.34–0.96)**	**0.034**	**1.65 (1.06–2.59)**	**0.027**	1.31 (0.74–2.32)	0.355
Control														
Vaccines (*n* = 38)	Reference	—	Reference	—	Reference	—	Reference	—	Reference	—	Reference	—	Reference	—
Drugs (*n* = 191)	1.75 (0.73–4.20)	0.214	0.15 (0.07–0.33)	**< 0.001**	**0.36 (0.17–0.78)**	**0.009**	1.48 (0.71–3.06)	0.293	**0.27 (0.12–0.59)**	**< 0.001**	1.74 (0.78–3.89)	0.177	0.84 (0.36–1.99)	0.694
Combined strategies (*n* = 165)	**2.60 (1.08–6.26)**	**0.033**	0.15 (0.07–0.33)	**< 0.001**	0.52 (0.24–1.12)	0.093	0.79 (0.37–1.67)	0.535	**0.45 (0.21–0.95)**	**0.037**	1.99 (0.88–4.48)	0.096	0.61 (0.25–1.49)	0.275
Presence of *C. perfringens*														
No (*n* = 223)	Reference	—	Reference	—	Reference	—	Reference	—	Reference	—	Reference	—	Reference	—
Yes (*n* = 171)	**2.40 (1.55–3.70)**	**< 0.001**	**1.80 (1.18–2.75)**	**0.006**	**2.20 (1.33–3.64)**	**0.002**	**2.62 (1.72-4.0)**	**< 0.001**	**1.82 (1.09–3.02)**	**0.022**	0.29 (0.85–1.96)	0.228	**1.85 (1.08–3.15)**	**0.024**

n – total number of samples, significant predictors in bold

*95% CI* 95% confidence interval, *OR* Odds ratio

**Table 9:** Multivariate analysis of putative flock-level risk factors associated with *Eimeria* species infection in broiler chickens from Guangdong province, China

Variables	*E. necatrix*		*E. tenella*		*E. brunetti*		*E. acervulina*		*E. maxima*		*E. mitis*		*E. praecox*	
	OR (95% CI)	* P*-value	OR (95% CI)	* P*-value	OR (95% CI)	* P*-value	OR (95% CI)	* P*-value	OR (95% CI)	* P*-value	OR (95% CI)	* P*-value	OR (95% CI)	* P*-value
Region														
Eastern (*n* = 87)	Reference	—	Reference	—	Reference	—	Reference	—	Reference	—	Reference	—	Reference	—
Western (*n* = 70)	2.34 (0.58–9.48)	0.233	1.30 (0.45–3.69)	0.629	1.02 (0.23–4.47)	0.981	1.13 (0.44–2.91)	0.799	0.60 (0.16–2.26)	0.449	0.53 (0.21–1.39)	0.199	0.37 (0.10–1.35)	0.134
Northern (*n* = 124)	**4.97 (1.19–20.73)**	**0.028**	2.90 (0.96–8.82)	0.060	1.81 (0.43–7.62)	0.417	0.56 (0.19–1.64)	0.291	1.22 (0.28–5.33)	0.795	0.53 (0.19–1.53)	0.241	0.74 (0.19–2.82)	0.654
Pearl River Delta (*n* = 113)	3.38 (0.99–11.55)	0.052	**2.48 (1.0-6.15)**	**0.050**	1.88 (0.59–6.01)	0.286	0.65 (0.28–1.51)	0.319	0.66 (0.23–1.91)	0.441	0.58 (0.25–1.35)	0.203	0.88 (0.32–2.45)	0.805
Age (weeks)														
Starter (0–4, *n* = 96)	Reference	—	Reference	—	Reference	—	Reference	—	Reference	—	Reference	—	Reference	—
Grower (4–8, *n* = 150)	2.08 (0.95–4.57)	0.068	1.35 (0.72–2.51)	0.351	**2.63 (1.15–6.04)**	**0.022**	0.63 (0.34–1.17)	0.147	**3.05 (1.23–7.59)**	**0.016**	**2.01 (1.08–3.73)**	**0.028**	**3.52 (1.44–8.62)**	**0.006**
Adult (> 8, *n* = 148)	**9.65 (4.45–20.94)**	**< 0.001**	0.89 (0.49–1.64)	0.714	**2.91 (1.31–6.44)**	**0.008**	0.78 (0.43–1.40)	0.408	**2.88 (1.23–6.77)**	**0.015**	1.28 (0.99–3.34)	0.054	2.12 (0.87–5.17)	0.097
Breed														
Indigenous crossbred (*n* = 190)	Reference	—	Reference	—	Reference	—	Reference	—	Reference	—	Reference	—	Reference	—
Indigenous (*n* = 204)	0.87 (0.49–1.54)	0.641	0.79 (0.48–1.31)	0.369	**0.48 (0.26–0.89)**	**0.021**	0.79 (0.48–1.32)	0.372	0.75 (0.36–1.54)	0.430	1.05 (0.65–1.70)	0.850	0.95 (0.50–1.79)	0.868
Farming														
Multi-layer cage (*n* = 50)	Reference	—	Reference	—	Reference	—	Reference	—	Reference	—	Reference	—	Reference	—
Ground floor (*n* = 344)	**0.34 (0.13–0.90)**	**0.031**	1.69 (0.68–4.24)	0.260	0.96 (0.29–3.12)	0.943	**2.63 (1.03–6.74)**	**0.043**	5.23 (0.92–29.93)	0.063	0.65 (0.27–1.54)	0.326	1.47 (0.47–4.60)	0.504
Drinking water														
Running water (*n* = 139)	Reference	—	Reference	—	Reference	—	Reference	—	Reference	—	Reference	—	Reference	—
Groundwater (*n* = 255)	1.11 (0.52–2.36)	0.792	0.84 (0.44–1.63)	0.609	0.44 (0.19–1.02)	0.055	1.67 (0.89–3.14)	0.111	0.52 (0.23–1.19)	0.124	1.04 (0.55–1.96)	0.898	0.93 (0.39–2.24)	0.875
Control														
Vaccines (*n* = 38)	Reference	—	Reference	—	Reference	—	Reference	—	Reference	—	Reference	—	Reference	—
Drugs (*n* = 191)	0.56 (0.11–2.78)	0.478	**0.09 (0.03–0.31)**	**< 0.001**	0.35 (0.08–1.48)	0.151	1.31 (0.43–3.99)	0.640	0.65 (0.16–2.55)	0.534	2.50 (0.78–7.94)	0.122	1.32 (0.33–5.35)	0.696
Combined strategies (*n* = 165)	1.11 (0.17–7.38)	0.918	**0.06 (0.01–0.25)**	**< 0.001**	0.51 (0.08–3.22)	0.472	0.71 (0.17–2.91)	0.636	0.74 (0.12–4.52)	0.745	3.71 (0.89–15.44)	0.072	0.84 (0.14–5.15)	0.846
Presence of *C. perfringens*														
No (*n* = 223)	Reference	—	Reference	—	Reference	—	Reference	—	Reference	—	Reference	—	Reference	—
Yes (*n* = 171)	**3.26 (1.96–5.43)**	**< 0.001**	**2.14 (1.36–3.36)**	**< 0.001**	**2.48 (1.45–4.23)**	**< 0.001**	**2.62 (1.69–4.06)**	**< 0.001**	**1.99 (1.16–3.42)**	**0.012**	1.27 (0.83–1.93)	0.278	**1.87 (1.08–3.24)**	**0.026**

n – total number of samples, significant predictors in bold

*95% CI* 95% confidence interval, *OR* Odds ratio

## Discussion

Coccidiosis poses a significant economical challenge for the global poultry industry. This study aimed to investigate the prevalence of *Eimeria* species in Guangdong province, filling a critical research gap [18–21]. The overall prevalence of coccidiosis in Guangdong (87.06%; 343/394) was found to be higher than that in other regions, such as Zhejiang province in China (30.7%; 95/310) [19], Shandong province in China (65.8%; 50/76) [20], Korean (75%; 291/388) [22], Serbia (59%; 59/100) [23], north India (28.5%; 171/600) [24], and southwestern Nigeria (41.3%; 2292/5544) [25]. The farm-level prevalence of *Eimeria* species in this study (98.88%; 88/89) was higher than that reported in Romania (92%; 11/12) [6]. The high prevalence of *Eimeria* species in Guangdong province can be attributed to the climatic conditions, characterized by increased temperature and humidity, which promote the propagation of *Eimeria* in broiler flocks. Our findings are consistent with previous reports from other tropical and subtropical regions and countries, including Anhui province in China (87.75%; 150/171) [21], two northern Indian states (81.03%; 47/58) [26] and Greece (85.7%; 36/42) [27]. However, higher prevalence rates were documented in Henan province (96.70%; 176/182) and Hubei province in China (97.79%; 133/136) [28], Colombia (96.3%; 236/245) [29], Australia (98%; 255/260) [30], Japan (91.9%; 33/37) [31], and northeastern Algeria (99.5%; 186/187) [32]. This variability can be attributed to differing climate conditions, seasonal variations, different terrains, and management practices in different regions and countries.

Seven distinct *Eimeria* species were identified within broiler farms in Guangdong province. The most prevalence species at the flock level were *E. acervulina* (36.55%; 144/394), *E. mitis* (35.28%; 139/394), *E. tenella* (34.01%; 134/394), and *E. necatrix* (30.96%; 122/394). It is well-known that the interactions between *Eimeria* species and crowing effects play a pivotal role in oocyst production [33]. *E. acervulina* and *E. tenella* exhibit higher productive potential, and in cases of mixed infection, *E. acervulina* tends to suppress the oocyst production of *E. necatrix*, *E. maxima*, and *E. brunetti* [34, 35]. Our study found that single-species infections were predominant at the flock level (37.31%; 147/394), with only 49.75% (196/394) of samples infected with two or more *Eimeria* species within a single fecal sample. The most common combination found was all seven *Eimeria* species (6.74%; 6/89), which differs from a previous report that found the most common combination to be *E. acervulina*, *E. maxima*, *E. necatrix*, and *E. praecox* (23.90%) in Pichincha and Santo Domingo de los Tsáchilas, Ecuador [36].

Univariate and multivariate analyses have identified several potential risk factors associated with the prevalence of *Eimeria* species. This study found that flocks with adult chickens faced a higher risk of *E. necatrix* infection (OR = 9.65, 95% CI: 4.45–20.94; *p* < 0.001) compared to starter chickens. This finding is consistent with previous reports, which have also suggested higher prevalence rates among adult birds compared to birds of other ages [37, 38]. However, it contrasts with studies by Lawal et al. [39] and Khursheed et al. [24], which reported that younger birds were more susceptible to infection than older birds. This discrepancy might be attributed to variations in the prevalence of *Eimeria* species. *E. necatrix* is known to have lower reproductive capabilities and is considered a ‘poor competitor’ compared to other species, which may explain its higher prevalence in older birds [40]. Notably, outbreaks due to *E. necatrix* predominantly occur in older birds aged 9–14 weeks [41]. The increase in epidemic *E. necatrix* prevalence observed in this study highlights the importance of improving preventative measures.

The association between geographical variation and elevated prevalence of coccidia has been reported in previous studies [42–44]. In this study, flocks from the Pearl River Delta had a higher risk of *E. tenella* occurrence (OR = 2.48, 95% CI: 1.0–6.15; *p* = 0.05) compared to those from eastern Guangdong. This could be due to the heavier rainfall (approximately 149 mm/year) and relatively lower humidity (approximately 57%) in the Pearl River Delta. These findings are consistent with a previous report by Waldenstedt et al. [45] which found that the sporulation of *Eimeria* oocysts was poorest under the conditions of high moisture content (62%), suggesting that oocyst sporulation may be more efficient in drier litter [40].

This study observed a lower risk of *E. tenella* infection in flocks that used anticoccidial drugs (OR = 0.09, 95% CI: 0.03–0.31; *p* < 0.001) or a combination of vaccines and anticoccidial drugs (OR = 0.06, 95% CI: 0.01–0.25; *p* < 0.001) compared to flocks that only used vaccines. This result is consistent with previous research, which found that oocyst shedding was significantly lower in medicated flocks compared to vaccinated flocks in chickens younger than 4 weeks (*p* < 0.05) [46]. Additionally, this study found a high prevalence of *E. brunetti* (19.80%; 78/394) in Guangdong, compared to a previous study in China (6.6%) [28], where no commercial vaccines containing *E. brunetti* are available. Given its classification as a highly pathogenic species, it may be necessary to include *E. brunetti* in vaccines in China. Furthermore, previous studies have shown that chickens raised in free-range systems have a higher occurrence of coccidiosis compared to those raised in cages [22, 28], as the main mode of transmission for sporulated oocysts of coccidia is through the fecal-oral route. In this study, a higher risk of *E. acervulina* infection (OR = 2.63, 95% CI: 1.03–6.74; *p* < 0.05) was found in ground-floored flocks compared to multi-layer caged flocks. However, ground-floored flocks were less likely to be positive for *E. necatrix* (OR = 0.34; 95% CI: 0.13–0.90; *p* < 0.05) compared to multi-layer caged flocks. The higher prevalence of coccidia in birds raised in multi-layer cages may be attributed to high bird density and suboptimal cage design or maintenance. Further studies with a larger sample size are needed to explore the prevalence of *Eimeria* in flocks using different farming methods.

In the present study, the occurrence of *C. perfringens* type A was significantly associated with the flock-level prevalence of *E. acervulina* (OR = 2.62, 95% CI: 1.69–4.06; *p* < 0.001), *E. necatrix* (OR = 3.26, 95% CI: 1.96–5.43; *p* < 0.001), *E. brunetti* (OR = 2.48, 95% CI: 1.45–4.23; *p* < 0.001), and *E. maxima* (OR = 1.99, 95% CI: 1.16–3.42; *p* < 0.05) compared to flocks where clostridia were not detected. Similarly, a previous study found that infection rates of *Eimeria* species were significantly associated with a history of clostridiosis on farms (OR = 2.6, 95% CI: 1.19–2.78; *p* = 0.006) [47]. The damage to the intestinal epithelium caused by coccidia creates an environment that allows for rapid replication and toxin production of *C. perfringens* [48]. In addition, experimental use of *C. perfringens* type A, and *E. acervulina* or *E. necatrix* has been shown to produce necrotic enteritis in chickens, with a mortality rate of 53% in chickens infected with *E. acervulina* before *C. perfringens* type A [48]. Under field conditions, coccidia can play a significant role in the occurrence of necrotic enteritis when there is a sufficient number of *C. perfringens* type A present [49].

## Conclusion

This study highlights the high prevalence of *Eimeria* species infections in broiler chickens across Guangdong province, China. The infection is widespread at both the farm and flock levels, with 98.88% (88/89) and 87.06% (343/394) of samples testing positive, respectively. The most common species found was *E. acervuline* in both farm and flock settings. Univariate and multivariate analysis revealed that geographical location, bird age, drinking water source, control methods, and the presence of *C. perfringens* type A were all associated with *Eimeria* species infection in chickens. Based on the identified risk factors, it is crucial to implement effective control strategies and management practices to reduce infections and minimize economic losses in poultry farming.

### Supplementary Information

**Supplementary Material 1.**

## Data Availability

The data that supporting the findings of this study, and the datasets used and/or analyzed during this study are available from the corresponding author on reasonable request.

## Abbreviations

*E. necatrix*: *Eimeria necatrix*
*E. tenella*: *Eimeria tenella*
*E. brunetti*: *Eimeria brunetti*
*E. acervulina*: *Eimeria acervulina*
*E. maxima*: *Eimeria maxima*
*E. mitis*: *Eimeria mitis*
*E. praecox*: *Eimeria praecox*
*C. perfringens*: *Clostridium perfringens*
PCR: Polymerase chain reaction
qPCR: Quantitative real-time polymerase chain reaction
ITS: Internal transcribed spacer
SCAR: Sequence characterized amplified region
OR: Odds ratio
CI: Confidence interval
