# Supplementary material for: Prevalence, geographic distribution and risk factors of Eimeria species on commercial broiler farms in Guangdong, China
Source: BMC Vet Res. 2024 May 3;20:171. doi: 10.1186/s12917-024-03990-4 (PMC11067301; doi:10.1186/s12917-024-03990-4)
Supplement: Supplementary file 1 — Supplementary Material 1. [file 12917_2024_3990_MOESM1_ESM.docx]

**Additional files:**

**Table S1. Questionnaire for veterinarians and farmers on different aspects of avian coccidiosis**

| **Category** | **Questions** | **Answers** |
| --- | --- | --- |
| Region | 1) In which city the farm is located?^a^ | 1. The 21 cities in Guangdong province, China |
| Type of production | 2)Which type of production for the flocks?^a^ | 1. a) Broiler flocks 2. b) Layer flocks 3. c) Multi-purpose flocks |
| Description of the flocks | 3) Age of birds^a^ | 1. The age of birds |
|  | 4) Breed of birds^a^ | 1. a) Indigenous 2. b) Indigenous crossbred 3. c) Non-indigenous crossbred |
|  | 5) Number of birds in the flocks^a^ | 1. a) Less than 10 birds/m^2^ 2. b) 10–16 birds/m^2^ 3. c) More than 16 birds/m^2^ |
|  | 6) Density of birds in the flocks^a^ | 1. a) Less than 5,000 birds 2. b) 5,000–10,000 birds 3. c) More than 10,000 birds |
| Management of the flocks | 7) Which type of farming do you apply?^a^ | 1. a) Ground floor 2. b) Multi-layer cage 3. c) Others |
|  | 8) What is the composition of litter?^a^ | 1. a) Wood shavings 2. b) Rice husk 3. c) Others |
|  | 9) Which type of drinking water do you apply?^a^ | 1. a) Running water 2. b) Groundwater 3. c) Others |
|  | 10) Which treatment measure of feces do you apply?^a^ | 1. a) Remove and non-ferment 2. b) Remove and ferment 3. c) Never remove 4. d) Others |
| Main diseases | 11) Select the two diseases affecting chicken flocks you are most concerned about^b,*^ | 1. a) Marek's disease 2. b) Coccidiosis   c) Avian influenza (bird flu)  d) virulent Newcastle disease (vND)  e) Salmonella  f) Mycoplasma  g) Necrotic enteritis  h) Others |
| Clinical signs, timing and risk factors for avian coccidiosis | 12) What are the two main signs by which you suspect coccidiosis?^b,*^ | a) Diarrhoea b) Diarrhoea and low body condition c) Diarrhoea and delay of growth d) Diarrhoea and immunosupresion e) Diarrhoea and mortality f) Others |
|  | 13) In which kind of flocks have you more frequently observed coccidiosis?^c,*^ | a) Flocks with large number of birds  b) Flocks with absence of cleaning and disinfection measures for broiler house  c) Flocks with presence of other diseases  d) In lots with problems of overcrowding and heterogeneity  e) Others |
|  | 14) In which period have you observed a greater incidence of coccidiosis?^b^ | a) 2–4 weeks  b) 4–8 weeks  c) At more than 8 weeks of age  d) There is no specific time point |
|  | 15) What are the two main signs by which you suspect necrotic enteritis? ^b,*^ | a) Diarrhea  b) Diarrhea and dehydration  c) Diarrhea and decrease in feed consumption  d) Diarrhea and severe depression  e) Others |
| Diagnosis of avian coccidiosis | 16) Do you perform microscopic detection to monitor coccidiosis?^b^ | a) Not routinely  b) Sporadically  c) Once or twice a year  d) Others |
|  | 17) Do you order species identification?^c^ | a) No, never  b) Only in the initial diagnosis  c) Yes, routinely |
| Control of avian coccidiosis | 18) Which have been your criteria for diagnosing and treating coccidiosis?^b^ | a) Up to 500 oocysts per gram of feces (opg)  b) 501–1,000 opg  c) 1,001–5,000 opg  d) >5,000 opg |
|  | 19) Which treatment/s do you use for coccidiosis?^b^ | a) Oral coccidiostats  b) Medicated feed  c) Vaccines  d) Combined treatment (vaccines and oral coccidiostats)  e) Others |
|  | 20) Which coccidiostat do you use?^c^ | a) Sulfachloropyrazine sodium  b) Nicarbazin  c) Maduramicin  d) Diclazuril  e) Narasin,  f) Others |
|  | 21) Which vaccine do you use?^c^ | a) Attenuated trivalent vaccine  b) Attenuated tetravalent vaccine  c) Coccivac^TM^  d) Others |

^*^Multiresponse question for which a maximum of 2 answers could be selected simultaneously.

^a^Question asked only to farmers.

^b^Question asked to both veterinarians and farmers.

^c^Question asked only to veterinarians.

**Table S2.** The using of anticoccidial agents in different cities

| **Region** | **Number of chicken farms** | **Number of samples** | **Application of coccidiostats** |
| --- | --- | --- | --- |
| Jieyang | 10 | 38 | Sulfachloropyrazine sodium |
| Chaozhou | 3 | 8 | Nicarbazin, maduramicin |
| Shantou | 5 | 23 | Sulfachloropyrazine sodium, narasin |
| Shanwei | 3 | 18 | Sulfachloropyrazine sodium, narasin |
| Zhanjiang | 3 | 12 | Nicarbazin, maduramicin, narasin |
| Maoming | 16 | 58 | Narasin, maduramicin |
| Qingyuan | 4 | 17 | Sulfachloropyrazine sodium |
| Shaoguan | 8 | 40 | Sulfachloropyrazine sodium, narasin |
| Heyuan | 4 | 26 | Nicarbazin, maduramicin |
| Meizhou | 2 | 4 | Nicarbazin, maduramicin, narasin |
| Yunfu | 6 | 37 | Sulfachloropyrazine sodium, narasin |
| Huizhou | 9 | 48 | Sulfachloropyrazine sodium, diclazuril |
| Zhaoqing | 5 | 24 | Sulfachloropyrazine sodium |
| Jiangmen | 4 | 18 | Unknown |
| Guangzhou | 5 | 19 | Sulfachloropyrazine sodium |
| Dongguan | 2 | 4 | Unknown |

**Table S3.** The results of species identification of *Eimeria* in Guangdong province

| **Region**^a^ | **Flock size** | **Age (days)** | **Breed**^b^ | **Type of farming**^c^ | **Type of drinking water**^d^ | **Treatment of manure**^e^ | **Cocciciostat given**^f^ | **Vaccination against coccidiosis**^g^ | ***C. perfringens* type A^h^** | **Clinical coccidiosis** | **Coccidia species**^i^ |
| --- | --- | --- | --- | --- | --- | --- | --- | --- | --- | --- | --- |
| JY1-1 | 5000 | 59 | TYC | GL | RW | N | N | Y | N | N | T |
| JY1-2 | 5000 | 59 | TYC | GL | RW | N | N | Y | N | N | T |
| JY1-3 | 5000 | 59 | TYC | GL | RW | N | N | Y | N | N | A T Ma Mi P |
| JY1-4 | 5000 | 59 | TYC | GL | RW | N | N | Y | Y | N | Mi |
| JY1-5 | 5000 | 59 | TYC | GL | RW | N | N | Y | Y | N | Mi |
| JY1-6 | 5000 | 59 | TYC | GL | RW | N | N | Y | Y | N | A |
| JY2-1 | 10000 | 42 | TYC | GL | RW | N | N | Y | N | N | A T P |
| JY2-2 | 10000 | 42 | TYC | GL | RW | N | N | Y | N | N | P |
| JY2-3 | 10000 | 42 | TYC | GL | RW | N | N | Y | N | N | T P |
| JY2-4 | 10000 | 42 | TYC | GL | RW | N | N | Y | N | N | A T Mi P |
| JY3-1 | 20000 | 54 | TYC | GL | RW | N | N | Y | Y | Y | A N T Ma |
| JY3-2 | 20000 | 54 | TYC | GL | RW | N | N | Y | N | N | nd |
| JY4-1 | 10000 | 60 | TYC | GL | RW | N | N | Y | Y | N | Ma |
| JY4-2 | 20000 | 60 | TYC | GL | RW | N | N | Y | Y | Y | B T Ma |
| JY4-3 | 20000 | 60 | TYC | GL | RW | N | N | Y | N | N | nd |
| JY5-1 | 10000 | 62 | TYC | GL | RW | N | N | Y | N | N | N |
| JY6-1 | 20000 | 22 | TYC | GL | RW | N | N | Y | N | N | T |
| JY6-2 | 20000 | 22 | TYC | GL | RW | N | N | Y | N | N | nd |
| JY7-1 | 15000 | 40 | TYC | GL | RW | N | N | Y | N | N | B |
| JY7-2 | 15000 | 40 | TYC | GL | RW | N | N | Y | Y | N | B N T Ma Mi |
| JY7-3 | 15000 | 40 | TYC | GL | RW | N | N | Y | N | N | T Ma |
| JY8-1 | 5000 | 20 | TYC | GL | RW | N | N | Y | N | N | T |
| JY8-2 | 5000 | 20 | TYC | GL | RW | N | N | Y | N | N | T |
| JY8-3 | 5000 | 20 | TYC | GL | RW | N | N | Y | N | N | A T |
| JY9-1 | 10000 | 65 | TYC | GL | RW | N | N | Y | Y | Y | N T |
| JY9-2 | 10000 | 65 | TYC | GL | RW | N | N | Y | Y | N | A T Ma |
| JY9-3 | 10000 | 65 | TYC | GL | RW | N | N | Y | N | N | T Ma |
| JY10-1 | 15000 | 51 | SBC | GL | RW | N | N | Y | N | N | B T |
| JY10-2 | 10000 | 51 | SBC | GL | RW | N | N | Y | N | N | B T Ma |
| JY10-3 | 10000 | 51 | SBC | GL | RW | N | N | Y | N | N | B T |
| JY10-4 | 5000 | 51 | SBC | GL | RW | N | N | Y | N | Y | A B T Ma |
| JY10-5 | 15000 | 51 | SBC | GL | RW | N | N | Y | Y | N | A B N T Mi P |
| JY10-6 | 10000 | 60 | SBC | GL | RW | N | N | Y | Y | N | B T Mi |
| JY10-7 | 10000 | 60 | SBC | GL | RW | N | N | Y | N | N | A B T Ma Mi |
| JY10-8 | 15000 | 60 | SBC | GL | RW | N | N | Y | N | N | A B T Ma |
| JY10-9 | 20000 | 60 | SBC | GL | RW | N | N | Y | Y | Y | A B N T Ma Mi P |
| JY10-10 | 20000 | 60 | SBC | GL | RW | N | N | Y | Y | N | A B N T P |
| JY10-11 | 20000 | 60 | SBC | GL | RW | N | N | Y | N | N | T Ma |
| CZ1-1 | 5000 | 27 | TYC | GL | RW | N | Y | N | N | N | T |

to be continued

| **District** | **Flock size** | **Age (days)** | **Breed** | **Type of farming** | **Type of drinking water** | **Treatment of manure** | **Cocciciostat given** | **Vaccination against coccidiosis** | ***C. perfringens* type A** | **Clinical coccidiosis** | **Coccidia species** |
| --- | --- | --- | --- | --- | --- | --- | --- | --- | --- | --- | --- |
| CZ1-2 | 5000 | 27 | TYC | GL | RW | N | Y | N | N | N | T Ma Mi |
| CZ1-3 | 5000 | 27 | TYC | GL | RW | N | Y | N | Y | N | A |
| CZ2-1 | 10000 | 28 | TYC | GL | RW | N | Y | N | N | N | T |
| CZ2-2 | 10000 | 28 | TYC | GL | RW | N | Y | N | N | N | Ma |
| CZ3-1 | 10000 | 25 | TYC | GL | RW | N | Y | N | Y | N | A |
| CZ3-2 | 10000 | 25 | TYC | GL | RW | N | Y | N | Y | N | A |
| CZ3-3 | 10000 | 25 | TYC | GL | RW | N | Y | N | Y | Y | A B |
| ST1-1 | 15000 | 37 | SBC | GL | GW | N | Y | N | N | N | T |
| ST1-2 | 15000 | 37 | SBC | GL | GW | N | Y | N | Y | N | B |
| ST2-1 | 15000 | 60 | SBC | GL | GW | N | Y | N | N | N | A Mi |
| ST2-2 | 15000 | 60 | SBC | GL | GW | N | Y | N | N | N | A B T Ma Mi |
| ST2-3 | 15000 | 60 | SBC | GL | GW | N | Y | N | Y | Y | A N T Ma Mi |
| ST2-4 | 15000 | 60 | SBC | GL | GW | N | Y | N | Y | N | A Ma Mi |
| ST2-5 | 15000 | 60 | SBC | GL | GW | N | Y | N | Y | N | Mi |
| ST2-6 | 15000 | 60 | SBC | GL | GW | N | Y | N | Y | N | Ma Mi |
| ST3-1 | 10000 | 34 | SBC | GL | GW | N | Y | N | N | N | A T Ma Mi P |
| ST3-2 | 10000 | 34 | SBC | GL | GW | N | Y | N | N | Y | A T Ma Mi P |
| ST3-3 | 10000 | 34 | SBC | GL | GW | N | Y | N | N | N | A |
| ST3-4 | 10000 | 34 | SBC | GL | GW | N | Y | N | Y | N | A T |
| ST3-5 | 10000 | 34 | SBC | GL | GW | N | Y | N | Y | Y | A N T Mi P |
| ST3-6 | 10000 | 34 | SBC | GL | GW | N | Y | N | Y | N | A |
| ST3-7 | 10000 | 34 | SBC | GL | GW | N | Y | N | Y | N | A Ma |
| ST3-8 | 10000 | 34 | SBC | GL | GW | N | Y | N | Y | N | A Ma |
| ST4-1 | 20000 | 25 | SBC | GL | GW | N | Y | N | N | N | A T Mi P |
| ST4-2 | 20000 | 25 | SBC | GL | GW | N | Y | N | Y | N | A |
| ST4-3 | 20000 | 25 | SBC | GL | GW | N | Y | N | Y | N | A |
| ST4-4 | 20000 | 25 | SBC | GL | GW | N | Y | N | Y | N | A |
| ST4-5 | 20000 | 25 | SBC | GL | GW | N | Y | N | Y | N | A |
| ST5-1 | 15000 | 57 | SBC | GL | GW | N | Y | N | N | N | Mi |
| ST5-2 | 15000 | 57 | SBC | GL | GW | N | Y | N | Y | N | Mi |
| SW1-1 | 5000 | 35 | SBC | GL | GW | N | Y | N | N | N | A Mi |
| SW1-2 | 5000 | 35 | SBC | GL | GW | N | Y | N | N | N | A |
| SW1-3 | 5000 | 35 | SBC | GL | GW | N | Y | N | N | N | A Mi P |
| SW1-4 | 5000 | 35 | SBC | GL | GW | N | Y | N | N | N | nd |
| SW1-5 | 5000 | 35 | SBC | GL | GW | N | Y | N | N | N | A P |
| SW1-6 | 5000 | 35 | SBC | GL | GW | N | Y | N | Y | N | Mi P |
| SW1-7 | 5000 | 35 | SBC | GL | GW | N | Y | N | Y | Y | B N P |
| SW2-1 | 10000 | 51 | SBC | GL | GW | N | Y | N | N | N | Mi P |
| SW2-2 | 10000 | 51 | SBC | GL | GW | N | Y | N | N | N | A Mi |
| SW2-3 | 10000 | 51 | SBC | GL | GW | N | Y | N | N | N | A Mi P |
| SW2-4 | 10000 | 51 | SBC | GL | GW | N | Y | N | N | N | P |

to be continued

| **District** | **Flock size** | **Age (days)** | **Breed** | **Type of farming** | **Type of drinking water** | **Treatment of manure** | **Cocciciostat given** | **Vaccination against coccidiosis** | ***C. perfringens* type A** | **Clinical coccidiosis** | **Coccidia species** |
| --- | --- | --- | --- | --- | --- | --- | --- | --- | --- | --- | --- |
| SW2-5 | 10000 | 51 | SBC | GL | GW | N | Y | N | Y | N | nd |
| SW2-6 | 10000 | 51 | SBC | GL | GW | N | Y | N | Y | N | Mi |
| SW3-1 | 15000 | 59 | SBC | GL | GW | N | Y | N | N | Y | A N T Mi P |
| SW3-2 | 15000 | 59 | SBC | GL | GW | N | Y | N | N | N | N P |
| SW3-3 | 15000 | 59 | SBC | GL | GW | N | Y | N | N | N | N |
| SW3-4 | 15000 | 59 | SBC | GL | GW | N | Y | N | Y | N | B |
| SW3-5 | 15000 | 59 | SBC | GL | GW | N | Y | N | Y | N | nd |
| ZJ1-1 | 20000 | 27 | TYC | GL | RW | N | Y | N | N | N | Mi |
| ZJ1-2 | 20000 | 27 | TYC | GL | RW | N | Y | N | Y | N | N T Ma P |
| ZJ1-3 | 20000 | 27 | TYC | GL | RW | N | Y | N | Y | N | nd |
| ZJ1-4 | 20000 | 27 | TYC | GL | RW | N | Y | N | Y | Y | A |
| ZJ2-1 | 10000 | 52 | TYC | GL | RW | N | Y | N | N | N | Mi |
| ZJ2-2 | 10000 | 52 | TYC | GL | RW | N | Y | N | Y | N | Mi |
| ZJ2-3 | 9000 | 23 | TYC | GL | RW | N | Y | N | Y | N | A B T Ma |
| ZJ2-4 | 9000 | 23 | TYC | GL | RW | N | Y | N | N | Y | A T Mi |
| ZJ2-5 | 9500 | 23 | TYC | GL | RW | N | Y | N | Y | N | A P |
| ZJ3-1 | 10000 | 23 | TYC | GL | RW | N | Y | N | Y | N | A |
| ZJ3-2 | 10000 | 23 | TYC | GL | RW | N | Y | N | N | N | A |
| ZJ3-3 | 10000 | 23 | TYC | GL | RW | N | Y | N | N | Y | A T Ma |
| MM1-1 | 10000 | 65 | TYC | GL | RW | N | Y | N | N | N | N |
| MM1-2 | 10000 | 65 | TYC | GL | RW | N | Y | N | Y | N | Mi |
| MM1-3 | 10000 | 65 | TYC | GL | RW | N | Y | N | Y | N | A B Ma |
| MM2-1 | 15000 | 25 | TYC | GL | RW | N | Y | N | Y | N | A |
| MM2-2 | 15000 | 25 | TYC | GL | RW | N | Y | N | N | N | A T |
| MM2-3 | 14500 | 25 | TYC | GL | RW | N | Y | N | N | Y | A T |
| MM2-4 | 15000 | 25 | TYC | GL | RW | N | Y | N | Y | N | nd |
| MM3-1 | 10000 | 27 | TYC | GL | RW | N | Y | N | Y | N | P |
| MM3-2 | 11000 | 27 | TYC | GL | RW | N | Y | N | Y | N | T |
| MM3-3 | 10500 | 27 | TYC | GL | RW | N | Y | N | N | N | nd |
| MM3-4 | 10000 | 27 | TYC | GL | RW | N | Y | N | N | N | A |
| MM4-1 | 10000 | 76 | TYC | GL | RW | N | Y | N | N | N | Mi |
| MM4-2 | 10000 | 76 | TYC | GL | RW | N | Y | N | N | N | A Ma Mi |
| MM4-3 | 10000 | 76 | TYC | GL | RW | N | Y | N | N | N | A |
| MM5-1 | 15000 | 90 | WCC | GL | GW | N | Y | N | N | N | Mi |
| MM5-2 | 15000 | 90 | WCC | GL | GW | N | Y | N | Y | N | A B T |
| MM6-1 | 15000 | 40 | TYC | GL | GW | N | Y | N | N | N | Mi |
| MM6-2 | 15000 | 40 | TYC | GL | GW | N | Y | N | Y | N | A Ma |
| MM7-1 | 20000 | 39 | TYC | GL | GW | N | Y | N | N | N | Mi |
| MM7-2 | 20000 | 39 | TYC | GL | GW | N | Y | N | Y | N | A T |
| MM7-3 | 20000 | 39 | TYC | GL | GW | N | Y | N | Y | N | A |

to be continued

| **District** | **Flock size** | **Age (days)** | **Breed** | **Type of farming** | **Type of drinking water** | **Treatment of manure** | **Cocciciostat given** | **Vaccination against coccidiosis** | ***C. perfringens* type A** | **Clinical coccidiosis** | **Coccidia species** |
| --- | --- | --- | --- | --- | --- | --- | --- | --- | --- | --- | --- |
| MM8-1 | 10000 | 68 | TYC | GL | GW | N | Y | N | N | N | A T |
| MM8-2 | 10000 | 68 | TYC | GL | GW | N | Y | N | N | N | nd |
| MM8-3 | 10000 | 68 | TYC | GL | GW | N | Y | N | N | N | N |
| MM8-4 | 10000 | 68 | TYC | GL | GW | N | Y | N | N | Y | B N Mi |
| MM8-5 | 10000 | 68 | TYC | GL | GW | N | Y | N | N | N | Mi P |
| MM8-6 | 10000 | 68 | TYC | GL | GW | N | Y | N | Y | N | A Ma |
| MM8-7 | 10000 | 68 | TYC | GL | GW | N | Y | N | Y | N | N T |
| MM9-1 | 20000 | 42 | TYC | GL | GW | N | Y | N | N | N | T |
| MM9-2 | 20000 | 42 | TYC | GL | GW | N | Y | N | N | N | A N |
| MM9-3 | 20000 | 42 | TYC | GL | GW | N | Y | N | Y | N | A |
| MM9-4 | 20000 | 42 | TYC | GL | GW | N | Y | N | Y | N | A T P |
| MM10-1 | 15000 | 67 | TYC | GL | GW | N | Y | N | N | N | N |
| MM10-2 | 15000 | 67 | TYC | GL | GW | N | Y | N | N | N | N |
| MM10-3 | 15000 | 67 | TYC | GL | GW | N | Y | N | N | N | A B |
| MM11-1 | 20000 | 32 | SBC | GL | RW | N | Y | Y | Y | Y | A N Ma Mi |
| MM11-2 | 20000 | 32 | SBC | GL | RW | N | Y | Y | Y | N | B T |
| MM11-3 | 20000 | 32 | SBC | GL | RW | N | Y | Y | Y | N | A T |
| MM11-4 | 20000 | 32 | SBC | GL | RW | N | Y | Y | Y | N | A Mi |
| MM11-5 | 15000 | 32 | SBC | GL | RW | N | Y | Y | N | N | Mi |
| MM12-1 | 15000 | 56 | SBC | GL | RW | N | Y | Y | Y | N | Mi |
| MM12-2 | 14500 | 56 | SBC | GL | RW | N | Y | Y | N | N | A N T |
| MM12-3 | 15000 | 56 | SBC | GL | RW | N | Y | Y | Y | N | A B N Ma |
| MM13-1 | 15000 | 90 | SBC | GL | RW | N | Y | Y | N | Y | B Mi |
| MM13-2 | 15000 | 90 | SBC | GL | RW | N | Y | Y | N | N | A |
| MM13-3 | 14800 | 90 | SBC | GL | RW | N | Y | Y | N | N | nd |
| MM13-4 | 15000 | 90 | SBC | GL | RW | N | Y | Y | Y | N | A B P |
| MM14-1 | 20000 | 90 | SBC | GL | RW | N | Y | Y | N | N | Mi |
| MM14-2 | 20000 | 90 | SBC | GL | RW | N | Y | Y | Y | N | N |
| MM14-3 | 20000 | 90 | SBC | GL | RW | N | Y | Y | N | N | N T |
| MM14-4 | 20000 | 90 | SBC | GL | RW | N | Y | Y | N | N | nd |
| MM15-1 | 19500 | 35 | SBC | GL | RW | N | Y | Y | Y | N | A B N Ma |
| MM15-2 | 19000 | 35 | SBC | GL | RW | N | Y | Y | Y | N | N |
| MM15-3 | 18500 | 35 | SBC | GL | RW | N | Y | Y | N | N | B Ma Mi |
| MM15-4 | 19000 | 35 | SBC | GL | RW | N | Y | Y | N | N | nd |
| MM15-5 | 19500 | 35 | SBC | GL | RW | N | Y | Y | N | N | Mi |
| MM16-1 | 20000 | 73 | SBC | GL | RW | N | Y | Y | Y | Y | B N Ma Mi |
| MM16-2 | 20000 | 73 | SBC | GL | RW | N | Y | Y | N | N | Mi P |
| QY1-1 | 18500 | 77 | QYPC | GL | RW | N | Y | Y | N | N | N |
| QY1-2 | 17500 | 75 | QYPC | GL | RW | N | Y | Y | Y | N | N |
| QY1-3 | 18000 | 66 | QYPC | GL | RW | N | Y | Y | Y | N | A B |

to be continued

| **District** | **Flock size** | **Age (days)** | **Breed** | **Type of farming** | **Type of drinking water** | **Treatment of manure** | **Cocciciostat given** | **Vaccination against coccidiosis** | ***C. perfringens* type A** | **Clinical coccidiosis** | **Coccidia species** |
| --- | --- | --- | --- | --- | --- | --- | --- | --- | --- | --- | --- |
| QY2-1 | 17500 | 22 | QYPC | GL | RW | N | Y | Y | N | N | Mi |
| QY2-2 | 17500 | 22 | QYPC | GL | RW | N | Y | Y | N | N | Mi |
| QY2-3 | 18000 | 22 | QYPC | GL | RW | N | Y | Y | Y | Y | N |
| QY2-4 | 18000 | 22 | QYPC | GL | RW | N | Y | Y | Y | N | N |
| QY3-1 | 15000 | 26 | QYPC | GL | RW | N | Y | Y | N | N | B |
| QY3-2 | 15000 | 26 | QYPC | GL | RW | N | Y | Y | N | N | Mi |
| QY3-3 | 15000 | 26 | QYPC | GL | RW | N | Y | Y | N | N | Mi |
| QY3-4 | 15000 | 26 | QYPC | GL | RW | N | Y | Y | N | N | Mi |
| QY3-5 | 15000 | 26 | QYPC | GL | RW | N | Y | Y | Y | N | A |
| QY4-1 | 22000 | 25 | QYPC | GL | RW | N | Y | Y | N | N | T |
| QY4-2 | 22000 | 25 | QYPC | GL | RW | N | Y | Y | N | N | nd |
| QY4-3 | 20000 | 25 | QYPC | GL | RW | N | Y | Y | N | N | B N Ma Mi |
| QY4-4 | 20000 | 25 | QYPC | GL | RW | N | Y | Y | N | N | N Ma |
| QY4-5 | 20000 | 25 | QYPC | GL | RW | N | Y | Y | N | N | nd |
| SG1-1 | 40000 | 60 | QYPC | GL | GW | N | Y | Y | N | N | N |
| SG1-2 | 20000 | 60 | QYPC | GL | GW | N | Y | Y | N | N | A B |
| SG1-3 | 25000 | 60 | QYPC | GL | GW | N | Y | Y | N | Y | A N |
| SG1-4 | 20000 | 60 | QYPC | GL | GW | N | Y | Y | Y | N | A N |
| SG1-5 | 22000 | 60 | QYPC | GL | GW | N | Y | Y | Y | N | A N |
| SG2-1 | 25000 | 63 | HYBC | GL | GW | N | Y | Y | N | N | A N Mi P |
| SG2-2 | 25000 | 63 | HYBC | GL | GW | N | Y | Y | N | N | N Mi |
| SG2-3 | 20000 | 63 | HYBC | GL | GW | N | Y | Y | Y | N | A |
| SG2-4 | 27000 | 33 | HYBC | GL | GW | N | Y | Y | Y | N | Mi |
| SG2-5 | 25000 | 33 | HYBC | GL | GW | N | Y | Y | Y | N | Mi |
| SG2-6 | 26000 | 33 | HYBC | GL | GW | N | Y | Y | Y | N | Mi |
| SG2-7 | 25000 | 33 | HYBC | GL | GW | N | Y | Y | Y | N | Mi |
| SG3-1 | 36000 | 38 | QYPC | GL | GW | N | Y | Y | N | N | B Mi |
| SG3-2 | 27000 | 38 | QYPC | GL | GW | N | Y | Y | N | N | Mi |
| SG3-3 | 26000 | 38 | QYPC | GL | GW | N | Y | Y | N | N | A Ma Mi |
| SG3-4 | 20000 | 38 | QYPC | GL | GW | N | Y | Y | N | N | A Ma Mi |
| SG3-5 | 28000 | 38 | QYPC | GL | GW | N | Y | Y | N | N | A Ma Mi |
| SG3-6 | 25000 | 38 | QYPC | GL | GW | N | Y | Y | Y | Y | A B N T |
| SG4-1 | 16000 | 34 | QYPC | GL | GW | N | Y | Y | N | N | Mi |
| SG4-2 | 20000 | 34 | QYPC | GL | GW | N | Y | Y | N | N | Mi |
| SG4-3 | 20000 | 34 | QYPC | GL | GW | N | Y | Y | N | N | Mi |
| SG4-4 | 20000 | 34 | QYPC | GL | GW | N | Y | Y | N | N | Mi |
| SG4-5 | 18000 | 34 | QYPC | GL | GW | N | Y | Y | Y | N | T Mi P |
| SG4-6 | 20000 | 34 | QYPC | GL | GW | N | Y | Y | Y | N | Mi |
| SG4-7 | 20000 | 34 | QYPC | GL | GW | N | Y | Y | Y | N | T Mi |
| SG4-8 | 20000 | 34 | QYPC | GL | GW | N | Y | Y | Y | N | N T Mi |

to be continued

| **District** | **Flock size** | **Age (days)** | **Breed** | **Type of farming** | **Type of drinking water** | **Treatment of manure** | **Cocciciostat given** | **Vaccination against coccidiosis** | ***C. perfringens* type A** | **Clinical coccidiosis** | **Coccidia species** |
| --- | --- | --- | --- | --- | --- | --- | --- | --- | --- | --- | --- |
| SG4-9 | 25000 | 34 | QYPC | GL | GW | N | Y | Y | Y | N | Mi P |
| SG4-10 | 25000 | 34 | QYPC | GL | GW | N | Y | Y | Y | N | N T Mi |
| SG5-1 | 36000 | 30 | QYPC | GL | GW | N | Y | Y | N | N | nd |
| SG5-2 | 36000 | 30 | QYPC | GL | GW | N | Y | Y | N | N | N T |
| SG6-1 | 27000 | 71 | QYPC | GL | GW | N | Y | Y | N | N | A N T Ma Mi |
| SG6-2 | 25000 | 71 | QYPC | GL | GW | N | Y | Y | N | N | nd |
| SG6-3 | 25000 | 71 | QYPC | GL | GW | N | Y | Y | N | Y | N T Ma |
| SG6-4 | 27000 | 71 | QYPC | GL | GW | N | Y | Y | N | N | nd |
| SG7-1 | 20000 | 71 | QYPC | GL | GW | N | Y | Y | N | N | N T |
| SG7-2 | 20000 | 71 | QYPC | GL | GW | N | Y | Y | N | N | nd |
| SG8-1 | 26000 | 71 | QYPC | GL | GW | N | Y | Y | Y | N | A N T |
| SG8-2 | 26000 | 71 | QYPC | GL | GW | N | Y | Y | Y | Y | A B N T P |
| SG8-3 | 25000 | 71 | QYPC | GL | GW | N | Y | Y | Y | N | N Ma |
| SG8-4 | 25000 | 71 | QYPC | GL | GW | N | Y | Y | Y | N | T Ma |
| HY1-1 | 15800 | 36 | SBC | MLC | GW | F | Y | N | N | N | Mi |
| HY1-2 | 15300 | 36 | SBC | MLC | GW | F | Y | N | N | N | A Mi |
| HY1-3 | 15000 | 36 | SBC | MLC | GW | F | Y | N | N | N | Ma Mi |
| HY1-4 | 15000 | 36 | SBC | MLC | GW | F | Y | N | N | N | A Ma |
| HY1-5 | 15600 | 36 | SBC | MLC | GW | F | Y | N | Y | N | A N T Mi |
| HY1-6 | 15000 | 36 | SBC | MLC | GW | F | Y | N | Y | N | T |
| HY1-7 | 15000 | 36 | SBC | MLC | GW | F | Y | N | Y | N | T Mi |
| HY2-1 | 7000 | 65 | SBC | MLC | GW | F | Y | N | N | N | N |
| HY2-2 | 7700 | 65 | SBC | MLC | GW | F | Y | N | N | N | N |
| HY2-3 | 7600 | 65 | SBC | MLC | GW | F | Y | N | Y | Y | N |
| HY2-4 | 7000 | 65 | SBC | MLC | GW | F | Y | N | Y | N | N Mi |
| HY2-5 | 7900 | 65 | SBC | MLC | GW | F | Y | N | Y | N | N P |
| HY2-6 | 7900 | 65 | SBC | MLC | GW | F | Y | N | Y | N | N T |
| HY3-1 | 7700 | 77 | SBC | MLC | GW | F | Y | N | N | N | N |
| HY3-2 | 7600 | 77 | SBC | MLC | GW | F | Y | N | N | N | N |
| HY3-3 | 7500 | 77 | SBC | MLC | GW | F | Y | N | N | N | Mi |
| HY4-1 | 15000 | 79 | SBC | MLC | GW | F | Y | N | N | N | A N |
| HY4-2 | 15000 | 79 | SBC | MLC | GW | F | Y | N | N | N | A B N Mi |
| HY4-3 | 15400 | 79 | SBC | MLC | GW | F | Y | N | N | N | N |
| HY4-4 | 15500 | 79 | SBC | MLC | GW | F | Y | N | N | N | N Mi |
| HY4-5 | 15000 | 79 | SBC | MLC | GW | F | Y | N | N | N | B N Mi P |
| HY4-6 | 15000 | 24 | SBC | MLC | GW | F | Y | N | Y | N | A Mi P |
| HY4-7 | 15400 | 24 | SBC | MLC | GW | F | Y | N | Y | N | N T |
| HY4-8 | 15000 | 24 | SBC | MLC | GW | F | Y | N | Y | N | A |
| HY4-9 | 15000 | 24 | SBC | MLC | GW | F | Y | N | Y | Y | B T Mi |
| HY4-10 | 15300 | 24 | SBC | MLC | GW | F | Y | N | Y | N | N |

to be continued

| **District** | **Flock size** | **Age (days)** | **Breed** | **Type of farming** | **Type of drinking water** | **Treatment of manure** | **Cocciciostat given** | **Vaccination against coccidiosis** | ***C. perfringens* type A** | **Clinical coccidiosis** | **Coccidia species** |
| --- | --- | --- | --- | --- | --- | --- | --- | --- | --- | --- | --- |
| MZ1-1 | 9000 | 26 | SBC | GL | RW | N | Y | N | N | N | A B T |
| MZ1-2 | 9500 | 26 | SBC | GL | RW | N | Y | N | N | N | nd |
| MZ2-1 | 10000 | 26 | SBC | GL | RW | N | Y | N | N | N | Ma |
| MZ2-2 | 10000 | 26 | SBC | GL | RW | N | Y | N | N | N | nd |
| YF1-1 | 10000 | 67 | SBC | GL | GW | N | Y | Y | N | N | T |
| YF1-2 | 9000 | 67 | SBC | GL | GW | N | Y | Y | N | N | N |
| YF1-3 | 10000 | 36 | SBC | GL | GW | N | Y | Y | N | N | nd |
| YF1-4 | 9600 | 36 | SBC | GL | GW | N | Y | Y | Y | N | A B N T Ma |
| YF1-5 | 10000 | 36 | SBC | GL | GW | N | Y | Y | N | N | nd |
| YF1-6 | 10000 | 67 | SBC | GL | GW | N | Y | Y | N | N | T |
| YF1-7 | 10000 | 67 | SBC | GL | GW | N | Y | Y | N | N | N T |
| YF2-1 | 10000 | 86 | TMBC | GL | GW | N | Y | Y | Y | Y | A B N T Mi P |
| YF2-2 | 10000 | 86 | TMBC | GL | GW | N | Y | Y | N | N | nd |
| YF2-3 | 10000 | 86 | TMBC | GL | GW | N | Y | Y | N | N | T |
| YF3-1 | 9000 | 72 | TMBC | GL | GW | N | Y | Y | Y | N | A N Ma |
| YF3-2 | 9000 | 72 | TMBC | GL | GW | N | Y | Y | N | N | N |
| YF3-3 | 9500 | 72 | TMBC | GL | GW | N | Y | Y | Y | N | N T Ma Mi |
| YF3-4 | 9000 | 72 | TMBC | GL | GW | N | Y | Y | N | N | nd |
| YF3-5 | 9000 | 72 | TMBC | GL | GW | N | Y | Y | Y | N | A B N T Ma Mi P |
| YF3-6 | 9000 | 72 | TMBC | GL | GW | N | Y | Y | Y | Y | B N Ma Mi |
| YF3-7 | 9600 | 39 | TMBC | GL | GW | N | Y | Y | N | N | nd |
| YF3-8 | 9000 | 39 | TMBC | GL | GW | N | Y | Y | N | N | N |
| YF3-9 | 10000 | 39 | TMBC | GL | GW | N | Y | Y | Y | N | B T P |
| YF4-1 | 15000 | 63 | TMBC | GL | GW | N | Y | Y | Y | Y | A B N T P |
| YF4-2 | 15000 | 63 | TMBC | GL | GW | N | Y | Y | N | N | A B N |
| YF4-3 | 14700 | 63 | TMBC | GL | GW | N | Y | Y | Y | N | A N T Ma Mi P |
| YF4-4 | 14500 | 63 | TMBC | GL | GW | N | Y | Y | N | N | B N |
| YF4-5 | 15000 | 63 | TMBC | GL | GW | N | Y | Y | N | N | T Ma |
| YF5-1 | 10000 | 50 | TMBC | GL | GW | N | Y | Y | N | N | nd |
| YF5-2 | 18000 | 50 | TMBC | GL | GW | N | Y | Y | N | N | T Ma |
| YF5-3 | 18700 | 50 | TMBC | GL | GW | N | Y | Y | Y | N | A B N T Mi P |
| YF6-1 | 19000 | 55 | TMBC | GL | GW | N | Y | Y | Y | N | N T Ma |
| YF6-2 | 20000 | 55 | TMBC | GL | GW | N | Y | Y | Y | N | N T P |
| YF6-3 | 20000 | 55 | TMBC | GL | GW | N | Y | Y | Y | Y | B N T Ma P |
| YF6-4 | 19000 | 24 | TMBC | GL | GW | N | Y | Y | N | N | nd |
| YF6-5 | 18600 | 24 | TMBC | GL | GW | N | Y | Y | N | N | T |
| YF6-6 | 19500 | 54 | TMBC | GL | GW | N | Y | Y | N | N | nd |
| YF6-7 | 20000 | 24 | TMBC | GL | GW | N | Y | Y | Y | N | B Mi P |
| YF6-8 | 19000 | 55 | TMBC | GL | GW | N | Y | Y | N | N | Ma |
| YF6-9 | 20000 | 55 | TMBC | GL | GW | N | Y | Y | N | N | nd |

to be continued

| **District** | **Flock size** | **Age (days)** | **Breed** | **Type of farming** | **Type of drinking water** | **Treatment of manure** | **Cocciciostat given** | **Vaccination against coccidiosis** | ***C. perfringens* type A** | **Clinical coccidiosis** | **Coccidia species** |
| --- | --- | --- | --- | --- | --- | --- | --- | --- | --- | --- | --- |
| YF6-10 | 20000 | 55 | TMBC | GL | GW | N | Y | Y | N | N | N T P |
| HZ1-1 | 9900 | 25 | XSC | GL | GW | F | Y | Y | N | N | B |
| HZ1-2 | 10000 | 25 | XSC | GL | GW | F | Y | Y | N | N | P |
| HZ1-3 | 10000 | 25 | XSC | GL | GW | F | Y | Y | N | N | Mi |
| HZ1-4 | 11000 | 55 | XSC | GL | GW | F | Y | Y | Y | N | B T P |
| HZ1-5 | 10000 | 55 | XSC | GL | GW | F | Y | Y | Y | N | B N P |
| HZ1-6 | 8500 | 55 | XSC | GL | GW | F | Y | Y | Y | N | P |
| HZ2-1 | 9000 | 51 | XSC | GL | GW | F | Y | Y | N | N | B Mi P |
| HZ2-2 | 9500 | 51 | XSC | GL | GW | F | Y | Y | N | N | P |
| HZ2-3 | 9900 | 51 | XSC | GL | GW | F | Y | Y | N | N | A N Mi |
| HZ2-4 | 10000 | 51 | XSC | GL | GW | F | Y | Y | Y | N | A Mi |
| HZ2-5 | 10000 | 51 | XSC | GL | GW | F | Y | Y | Y | N | Mi |
| HZ3-1 | 8000 | 26 | XSC | GL | GW | F | Y | Y | N | N | Mi |
| HZ3-2 | 8000 | 26 | XSC | GL | GW | F | Y | Y | N | Y | A B Ma Mi |
| HZ3-3 | 8600 | 26 | XSC | GL | GW | F | Y | Y | N | N | Mi |
| HZ3-4 | 8500 | 26 | XSC | GL | GW | F | Y | Y | Y | N | A |
| HZ3-5 | 9000 | 26 | XSC | GL | GW | F | Y | Y | Y | N | Mi |
| HZ4-1 | 11000 | 24 | XSC | GL | GW | F | Y | Y | N | N | A |
| HZ4-2 | 10000 | 24 | XSC | GL | GW | F | Y | Y | N | N | Mi |
| HZ4-3 | 10000 | 24 | XSC | GL | GW | F | Y | Y | Y | N | T |
| HZ4-4 | 10500 | 24 | XSC | GL | GW | F | Y | Y | Y | N | A |
| HZ4-5 | 10000 | 24 | XSC | GL | GW | F | Y | Y | Y | N | T |
| HZ5-1 | 10000 | 28 | XSC | GL | GW | F | Y | Y | N | N | A N Mi |
| HZ5-2 | 11000 | 28 | XSC | GL | GW | F | Y | Y | N | N | A |
| HZ5-3 | 11000 | 28 | XSC | GL | GW | F | Y | Y | N | N | nd |
| HZ5-4 | 10000 | 28 | XSC | GL | GW | F | Y | Y | Y | N | A T |
| HZ5-5 | 10500 | 28 | XSC | GL | GW | F | Y | Y | Y | Y | N T Mi |
| HZ6-1 | 8000 | 17 | XSC | GL | GW | F | Y | Y | N | N | A |
| HZ6-2 | 8500 | 17 | XSC | GL | GW | F | Y | Y | N | N | Mi |
| HZ6-3 | 8500 | 17 | XSC | GL | GW | F | Y | Y | Y | N | A |
| HZ6-4 | 8600 | 17 | XSC | GL | GW | F | Y | Y | Y | N | A |
| HZ7-1 | 10000 | 45 | XSC | GL | GW | F | Y | Y | N | N | N Ma |
| HZ7-2 | 10000 | 45 | XSC | GL | GW | F | Y | Y | N | N | nd |
| HZ7-3 | 10500 | 45 | XSC | GL | GW | F | Y | Y | Y | Y | A N T Ma |
| HZ7-4 | 10500 | 45 | XSC | GL | GW | F | Y | Y | Y | N | nd |
| HZ8-1 | 10000 | 48 | XSC | GL | GW | F | Y | Y | Y | N | A N T Ma Mi |
| HZ8-2 | 9800 | 48 | XSC | GL | GW | F | Y | Y | N | N | nd |
| HZ8-3 | 9000 | 48 | XSC | GL | GW | F | Y | Y | N | N | N T |
| HZ8-4 | 10000 | 48 | XSC | GL | GW | F | Y | Y | N | Y | A T Ma Mi |
| HZ8-5 | 9000 | 48 | XSC | GL | GW | F | Y | Y | N | N | nd |

to be continued

| **District** | **Flock size** | **Age (days)** | **Breed** | **Type of farming** | **Type of drinking water** | **Treatment of manure** | **Cocciciostat given** | **Vaccination against coccidiosis** | ***C. perfringens* type A** | **Clinical coccidiosis** | **Coccidia species** |
| --- | --- | --- | --- | --- | --- | --- | --- | --- | --- | --- | --- |
| HZ8-6 | 9000 | 48 | XSC | GL | GW | F | Y | Y | Y | N | A B T Mi |
| HZ8-7 | 9000 | 48 | XSC | GL | GW | F | Y | Y | Y | N | A B T Ma |
| HZ8-8 | 8600 | 48 | XSC | GL | GW | F | Y | Y | N | N | nd |
| HZ8-9 | 9000 | 48 | XSC | GL | GW | F | Y | Y | N | N | B N Mi |
| HZ8-10 | 9000 | 48 | XSC | GL | GW | F | Y | Y | N | N | nd |
| HZ8-11 | 8700 | 48 | XSC | GL | GW | F | Y | Y | Y | Y | B N T Ma Mi P |
| HZ9-1 | 10500 | 43 | XSC | GL | GW | F | Y | Y | N | N | T Ma |
| HZ9-2 | 10000 | 43 | XSC | GL | GW | F | Y | Y | N | N | nd |
| HZ9-3 | 10000 | 43 | XSC | GL | GW | F | Y | Y | Y | N | B N T Ma |
| ZQ1-1 | 11500 | 58 | XHC | MLC | GW | F | Y | N | N | N | N |
| ZQ1-2 | 11000 | 58 | XHC | MLC | GW | F | Y | N | N | Y | B |
| ZQ1-3 | 13000 | 58 | XHC | MLC | GW | F | Y | N | N | N | Mi |
| ZQ1-4 | 11000 | 58 | XHC | MLC | GW | F | Y | N | Y | N | N |
| ZQ1-5 | 13000 | 58 | XHC | MLC | GW | F | Y | N | Y | N | N |
| ZQ2-1 | 13000 | 26 | XHC | MLC | GW | F | Y | N | N | N | Mi |
| ZQ2-2 | 11000 | 26 | XHC | MLC | GW | F | Y | N | N | N | A N T |
| ZQ2-3 | 11500 | 26 | XHC | MLC | GW | F | Y | N | N | N | N T |
| ZQ2-4 | 11000 | 26 | XHC | MLC | GW | F | Y | N | Y | N | A N T Mi |
| ZQ2-5 | 11000 | 26 | XHC | MLC | GW | F | Y | N | Y | N | A T |
| ZQ3-1 | 12000 | 31 | XHC | MLC | GW | F | Y | N | N | N | B P |
| ZQ3-2 | 11500 | 31 | XHC | MLC | GW | F | Y | N | N | Y | B |
| ZQ3-3 | 10500 | 31 | XHC | MLC | GW | F | Y | N | Y | N | A Mi P |
| ZQ4-1 | 11000 | 45 | XHC | MLC | GW | F | Y | N | N | N | nd |
| ZQ4-2 | 11000 | 45 | XHC | MLC | GW | F | Y | N | N | N | T |
| ZQ4-3 | 12000 | 45 | XHC | MLC | GW | F | Y | N | N | N | A T P |
| ZQ4-4 | 11500 | 45 | XHC | MLC | GW | F | Y | N | Y | N | Mi |
| ZQ4-5 | 11000 | 45 | XHC | MLC | GW | F | Y | N | Y | N | Mi |
| ZQ4-6 | 11000 | 45 | XHC | MLC | GW | F | Y | N | Y | N | Mi |
| ZQ5-1 | 8700 | 36 | HYBC | MLC | GW | F | Y | N | Y | N | N T P |
| ZQ5-2 | 9000 | 36 | HYBC | MLC | GW | F | Y | N | Y | Y | B N T Mi P |
| ZQ5-3 | 8600 | 36 | HYBC | MLC | GW | F | Y | N | N | N | nd |
| ZQ5-4 | 8500 | 36 | HYBC | MLC | GW | F | Y | N | N | N | B N T |
| ZQ5-5 | 8500 | 36 | HYBC | MLC | GW | F | Y | N | N | N | nd |
| JM1-1 | 13000 | 72 | TYC | GL | GW | F | Y | N | N | N | A N Mi |
| JM1-2 | 10000 | 72 | TYC | GL | GW | F | Y | N | N | N | N Mi |
| JM1-3 | 12000 | 72 | TYC | GL | GW | F | Y | N | Y | N | N Mi |
| JM1-4 | 11000 | 72 | TYC | GL | GW | F | Y | N | Y | N | A Mi |
| JM1-5 | 10000 | 72 | TYC | GL | GW | F | Y | N | Y | N | A N Mi |
| JM1-6 | 11000 | 72 | TYC | GL | GW | F | Y | N | Y | N | N Mi |
| JM2-1 | 23000 | 57 | WCC | GL | GW | F | Y | N | N | N | A |

to be continued

| **District** | **Flock size** | **Age (days)** | **Breed** | **Type of farming** | **Type of drinking water** | **Treatment of manure** | **Cocciciostat given** | **Vaccination against coccidiosis** | ***C. perfringens* type A** | **Clinical coccidiosis** | **Coccidia species** |
| --- | --- | --- | --- | --- | --- | --- | --- | --- | --- | --- | --- |
| JM2-2 | 22000 | 57 | WCC | GL | GW | F | Y | N | N | N | nd |
| JM2-3 | 20000 | 57 | WCC | GL | GW | F | Y | N | Y | N | A |
| JM2-4 | 20000 | 57 | WCC | GL | GW | F | Y | N | Y | N | A N Mi P |
| JM2-5 | 20000 | 57 | WCC | GL | GW | F | Y | N | Y | N | A Ma Mi |
| JM3-1 | 9300 | 23 | SBC | GL | GW | F | Y | N | Y | N | nd |
| JM3-2 | 9500 | 23 | SBC | GL | GW | F | Y | N | Y | N | A B |
| JM3-3 | 9000 | 23 | SBC | GL | GW | F | Y | N | Y | N | P |
| JM4-1 | 9000 | 37 | SBC | GL | GW | F | Y | N | Y | Y | A N T |
| JM4-2 | 9500 | 37 | SBC | GL | GW | F | Y | N | N | N | nd |
| JM4-3 | 9500 | 37 | SBC | GL | GW | F | Y | N | N | N | T |
| JM4-4 | 9000 | 37 | SBC | GL | GW | F | Y | N | N | N | nd |
| GZ1-1 | 15000 | 25 | SBC | GL | RW | N | Y | N | N | N | nd |
| GZ1-2 | 15000 | 25 | SBC | GL | RW | N | Y | N | N | N | T |
| GZ1-3 | 15000 | 25 | SBC | GL | RW | N | Y | N | N | N | T |
| GZ1-4 | 15000 | 25 | SBC | GL | RW | N | Y | N | N | N | nd |
| GZ2-1 | 10000 | 70 | SBC | GL | RW | N | Y | N | Y | N | A B N T Ma Mi |
| GZ2-2 | 10000 | 70 | SBC | GL | RW | N | Y | N | Y | N | T Mi P |
| GZ2-3 | 10000 | 70 | SBC | GL | RW | N | Y | N | N | N | nd |
| GZ2-4 | 10000 | 70 | SBC | GL | RW | N | Y | N | Y | Y | A N T Ma |
| GZ2-5 | 10000 | 70 | SBC | GL | RW | N | Y | N | N | N | T N P |
| GZ3-1 | 20000 | 69 | SBC | GL | RW | N | Y | N | N | N | nd |
| GZ3-2 | 20000 | 69 | SBC | GL | RW | N | Y | N | N | N | A B T Mi P |
| GZ3-3 | 20000 | 69 | SBC | GL | RW | N | Y | N | N | N | A B P |
| GZ3-4 | 20000 | 69 | SBC | GL | RW | N | Y | N | N | N | B |
| GZ4-1 | 15000 | 59 | SBC | GL | RW | N | Y | N | Y | N | A B T Ma P |
| GZ4-2 | 15000 | 59 | SBC | GL | RW | N | Y | N | N | N | A |
| GZ4-3 | 15000 | 59 | SBC | GL | RW | N | Y | N | Y | Y | A B N T Ma |
| GZ5-1 | 15000 | 63 | SBC | GL | RW | N | Y | N | Y | N | N T P |
| GZ5-2 | 15000 | 63 | SBC | GL | RW | N | Y | N | Y | N | B N T Mi P |
| GZ5-3 | 15000 | 63 | SBC | GL | RW | N | Y | N | Y | N | B N T |
| DG1-1 | 20000 | 65 | SBC | GL | RW | N | Y | N | Y | N | A B N T Ma Mi |
| DG1-2 | 20000 | 65 | SBC | GL | RW | N | Y | N | Y | N | A N T Mi |
| DG2-1 | 20000 | 65 | SBC | GL | RW | N | Y | N | N | N | nd |
| DG2-2 | 20000 | 65 | SBC | GL | RW | N | Y | N | N | N | nd |

^a^Place name abbreviations: JY, Jieyang; CZ, Chaozhou; ST, Shantou; SW, Shanwei; ZJ, Zhanjiang; MM, Maoming; QY, Qingyuan; SG, Shaoguan; HY, Heyuan; MZ, Meizhou; YF, Yunfu; HZ, Huizhou; ZQ, Zhaoqing; JM, Jiangmen; GZ, Guangzhou; DG, Dongguan

^b^Breed name abbreviations: TYC, Three-yellow chicken; SBC, Spotted-brown chicken; WCC, Wenchang chicken; QYPC, Qingyuan partridge chicken; HYBC, Huiyang bearded chicken; TMBC, Tianma-black chicken; XSC, Xueshan chicken; XHC, Xinghua chicken

^c^Type of farming abbreviations: GL, Ground floor; MLC, Multi-layer cage

^d^Type of water source abbreviations: RW, Running water; GW, Groundwater

^e^Treatment of manure abbreviations: N, non-fermentation; F, fermentation

^f^Cocciciostat given abbreviations: N, not given; Y, given

^g^Vaccination against coccidiosis abbreviations: N, non-vaccination; Y, vaccination

^h^Clinical NE occurrence abbreviations: N, no occurrence; Y, occurrence

^i^Coccidia species abbreviations: A, *Eimeria acervulina*; B, *E. brunetti*; N, *E. necatrix*, T, *E. tenella*; Ma, *E. maxima*; Mi, *E. mitis*; P, *Eimeria praecox*; nd, not detected


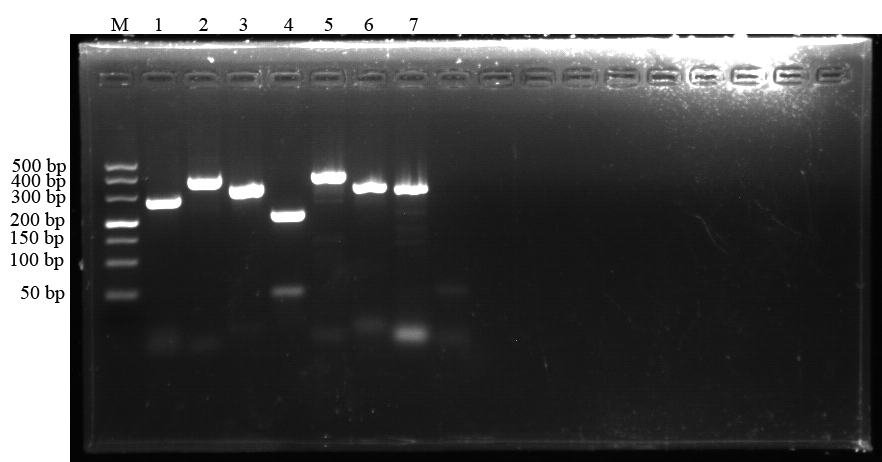


Figure S1. Gel electrophoresis of PCR products amplified from chicken fecal samples. Lane M: DNA ladder marker; Lane 1: PCR products from fecal samples with *E. tenella*; Lane 2: PCR products from fecal samples with *E. praecox*; Lane 3: PCR products from fecal samples with *E. mitis*; Lane 4: PCR products from fecal samples with *E. maxima*; Lane 5: PCR products from fecal samples with *E. necatrix*; Lane 6: PCR products from fecal samples with *E. acervulina*; Lane 7: PCR products from fecal samples with *E. brunetti.*
